# Supplementary material for: A hybrid simulation model to study the impact of combined interventions on Ebola epidemic
Source: PLoS One. 2021 Jul 6;16(7):e0254044. doi: 10.1371/journal.pone.0254044 (PMC8259970; doi:10.1371/journal.pone.0254044)
Supplement: S1 File — (DOCX) [file pone.0254044.s001.docx]

S1 File. Transitions and expressions in the hybrid model.

The description and valuation of the parameters used in this file are provided in Table1 and Table2 in our paper.

Transitions in the Macro level:

| Stock | Flow | Expressions | Flow description |
| --- | --- | --- | --- |
| $S_{G}$ | $S_{G}$→ E | $\left( \frac{\left( I\beta_{I_{\mathrm{NHCW}}}+H\beta_{H_{\mathrm{NHCW}}}+D\beta_{D_{\mathrm{NHCW}}} \right)}{N} \right)S_{G}$ | Infection of the general population by infected, hospitalized, and dead but not buried individuals |
| $S_{HCW}$ | $S_{HCW}$→ E | the daily new exposed cases for healthcare workers | Infection of healthcare workers by ABM output |
| E | E → I | σE | Be infected after the incubation period |
| I | I → H | αI | infectious individuals transmit to hospital |
|  | I → R | $\left( 1-\delta_{1} \right)\gamma I$ | Infectious individuals recover from disease |
|  | I → D | $\delta_{1}\gamma I$ | Infectious individuals die before hospitalization |
| H | H → R | ${(1-\delta}_{2})\gamma_{H}H$ | Hospitalized individuals recover from disease |
|  | H → D | $\delta_{2}\gamma_{H}H$ | Hospitalized individuals become dead |
| D | D → B | $\gamma_{D}D$ | Burial/isolation for dead individuals |

At the beginning of simulation, the whole population was considered susceptible. The size of each stock (sub-population) were estimated by the following differential equations based on the transitions in the Macro level :

$$\frac{dS_{G}}{\mathrm{dt}}=-\left( \frac{\left( I\beta_{I_{\mathrm{NHCW}}}+H\beta_{H_{\mathrm{NHCW}}}+D\beta_{D_{\mathrm{NHCW}}} \right)}{N} \right)S_{G}$$

$$\frac{dS_{\mathrm{HCW}}}{\mathrm{dt}}=-the daily new exposed cases for healthcare workers (decrease per day)$$

$$\frac{\mathrm{dE}}{\mathrm{dt}}=\frac{\left( I\beta_{I_{\mathrm{NHCW}}}+H\beta_{H_{\mathrm{NHCW}}}+D\beta_{D_{\mathrm{NHCW}}} \right)}{N}S_{G}+ \frac{\left( I\beta_{I_{\mathrm{HCW}}}+H\beta_{H_{\mathrm{HCW}}}+D\beta_{D_{\mathrm{HCW}}} \right)}{N}\left( S_{\mathrm{HCW}}+V_{1} \right)-\sigma E+the daily new exposed cases for healthcare workers (increase per day)$$

$$\frac{\mathrm{dI}}{\mathrm{dt}}=\sigma E-\alpha I-\delta_{1}\gamma I-\left( 1-\delta_{1} \right)\gamma I$$

$$\frac{\mathrm{dH}}{\mathrm{dt}}=\alpha I{-(1-\delta}_{2})\gamma_{H}H-\delta_{2}\gamma_{H}H$$

$$\frac{\mathrm{dR}}{\mathrm{dt}}=\left( 1-\delta_{1} \right)\gamma I+{(1-\delta}_{2})\gamma_{H}H$$

$$\frac{\mathrm{dD}}{\mathrm{dt}}=\delta_{1}\gamma I+\delta_{2}\gamma_{H}H-\gamma_{D}D$$

$$\frac{\mathrm{dB}}{\mathrm{dt}}=\gamma_{D}D$$

$$S_{G}\left( 0 \right)=N$$

$$S_{HCW}\left( 0 \right)=HCW$$

E(0)= I(0)= H(0)= D(0)= R(0)= F(0)=0

Transitions in the Micro level (Individual):

| Start State | End state | Transition rule |
| --- | --- | --- |
| start | working at health facility | 70% possibility^[1]^ |
|  | not working | 30% possibility |
| working at health facility | initial Ebola education(P1) | 1.time delay: 15 to 20 days  2.10% possibility^[1]^ |
|  | novice | 1.time delay: 15 to 20 days  2.90% possibility^[1]^ |
| not working | initial Ebola education(P1) | 10% possibility^[1]^ |
|  | novice | 60% possibility^[1]^ |
|  | not working | those who do not transfer to “novie” or “initial Ebola education” |
| initial Ebola education | not trained | 1.time delay: 15 to 20 days  2.those who do not transfer to “Educated training” |
|  | educated training(P2) | 1.time delay: 15 to 20 days  2.90% possibility |
| novice | to be trained(P3) | 1.time delay: 15 to 20 days  2.P3 possibility |
|  | uneducated | 1.time delay: 15 to 20 days  2.those who do not transfer to “to be trained” |
| not trained | unprotected | at rate of 0.1 person per day |
| educated training | trained | time delay: 3 days^[2]^ |
| to be trained | training | at rate of 5 person per day^[2]^ |
| training | trained | time delay: 5 days^[2]^ |
| uneducated | unprotected | at rate of 0.1 person per day |
| unprotected | exposed | time delay: 2 to 21 days |
| trained | V1 | depend on the coverage of vaccination |
|  | trained | those who do not turn to “V1” |
|  | exposed | at the possibility of  $\frac{\left( I\beta_{\mathrm{IHCW}}+{H\beta}_{\mathrm{HHCW}}+{D\beta}_{\mathrm{DHCW}} \right)}{N}$ |
| V1 | V2 | time delay: 7 days |
|  | exposed | at the possibility of  $\frac{\left( I\beta_{\mathrm{IHCW}}+{H\beta}_{\mathrm{HHCW}}+{D\beta}_{\mathrm{DHCW}} \right)}{N}$ |
| V2 | trained | time delay: 180 days |
|  | exposed | at the possibility of  $\frac{\left( I\beta_{\mathrm{IHCW}}+{H\beta}_{\mathrm{HHCW}}+{D\beta}_{\mathrm{DHCW}} \right)}{N}$ |

Here, we used the daily new exposed cases for healthcare workers in the micro level model to simulate the flow between $S_{HCW}$ and E.

| Vaccination rate | P3 | | | | | |
| --- | --- | --- | --- | --- | --- | --- |
|  | 0.95 | 0.96 | 0.97 | 0.98 | 0.99 | 1 |
| 0 | 14452(8650-16310) | 9838（8252-14009） | 8668（5803-11456） | 7360（1930-9008） | 5377（1700-7831） | 4206（559-4655） |
| Proportion of cases averted vs no vaccination | 0 | 31.93%（3.07%-42.90%） | 40.02%（20.73%-59.85%） | 49.07%（37.67%-86.65%） | 62.79%（45.81%-88.23%） | 70.90%（67.78%-96.13%） |
| 0.1 | 5475（3907-6041） | 4730（2410-5885） | 3771（1695-4009） | 2837（1117-3960） | 2035（540-3024） | 1131（162-1998） |
| Proportion of cases averted vs no vaccination | 62.11%（58.20%-72.96%） | 67.27%（59.28%-83.32%） | 73.91%（72.26%-88.27%） | 80.37%（72.60%-92.26%） | 85.92%（79.08%-96.26%） | 92.17%（86.17%-98.88%） |
| 0.3 | 5291（3890-6186） | 4396（2275-5966） | 3345（1422-3938） | 2624（951-2845） | 2045（554-2721） | 1199（180-1995） |
| Proportion of cases averted vs no vaccination | 63.39%（57.20%-73.06%） | 69.58%（58.72%-84.23%） | 76.85%（72.74%-90.16%） | 81.84%（80.30%-93.40%） | 85.85%（81.16%-96.15%） | 91.70%（86.18%-98.75%） |
| 0.5 | 5009（3853-5805） | 4258（2068-4527） | 3770（1487-4049） | 2625（695-2831） | 1966（230-2648） | 1345（111-2090） |
| Proportion of cases averted vs no vaccination | 65.34%（59.83%-73.34%） | 70.54%（68.68%-85.69%） | 73.91%（71.98%-89.71%） | 81.84%（80.41%-95.19%） | 86.40%（81.68%-98.41%） | 90.69%（85.54%-99.23%） |

Table 1. Impact of combined interventions on cumulative Ebola cases and the proportion of cases averted versus no vaccination scenarios.

| Vaccination rate | P3 | | | | | |
| --- | --- | --- | --- | --- | --- | --- |
|  | 0.95 | 0.96 | 0.97 | 0.98 | 0.99 | 1 |
| 0 | 4041(2734-5851) | 2754（2476-4350） | 2398（1931-4003） | 2034（1428-2849） | 1480（788-2396） | 1164（441-1530） |
| Proportion of cases averted vs no vaccination | 0 | 31.85%（0%-38.78%） | 40.66%（1.11%-52.30%） | 49.67%（29.62%-64.72%） | 63.38%（40.81%-80.53%） | 71.20%（62.20%-89.11%） |
| 0.1 | 1622（1320-2101） | 1399（1040-1773） | 1115（841-1530） | 841（617-1225） | 601（408-951） | 333（189-533） |
| Proportion of cases averted vs no vaccination | 59.86%（48.10%-67.39%） | 65.38%（56.20%-74.31%） | 72.41%（62.20%-79.22%） | 79.19%（69.73%-84.56%） | 85.10%（76.51%-89.92%） | 91.76%（87.67%-95.33%） |
| 0.3 | 1569（1222-2160） | 1308（1061-1800） | 992（763-1459） | 774（555-1177） | 603（396-843） | 353（123-499） |
| Proportion of cases averted vs no vaccination | 61.17%（46.64%-69.81%） | 67.63%（55.53%-73.79%） | 75.45%（63.90%-81.12%） | 80.85%（70.87%-86.27% | 85.08%（79.14%-90.20%） | 91.26%（87.65%-96.96%） |
| 0.5 | 1484（1203-2111） | 1261（962-1692） | 1019（785-1340） | 778（556-1120） | 581（381-833） | 330（172-510） |
| Proportion of cases averted vs no vaccination | 63.28%（47.76%-70.23%） | 68.79%（58.13%-76.19%） | 74.78%（66.84%-80.57%） | 80.75%（72.28%-86.24%） | 85.62%（79.39%-90.57） | 91.83%（87.38%-95.74%） |

Table 2. Impact of combined interventions on cumulative Ebola deaths and the proportion of deaths averted versus no vaccination scenarios.

[1] Annan A A, Yar D D, Owusu M, et al. Health care workers indicate ill preparedness for Ebola Virus Disease outbreak in Ashanti Region of Ghana[J]. BMC Public Health, 2017, 17(1): 1-7.

[2] Jones-Konneh TEC, Murakami A, Sasaki H, Egawa S. Intensive Education of Health Care Workers Improves the Outcome of Ebola Virus Disease: Lessons Learned from the 2014 Outbreak in Sierra Leone. Tohoku J Exp Med. 2017 Oct;243(2):101-105.
