# Supplementary material for: A hybrid simulation model to study the impact of combined interventions on Ebola epidemic
Source: PLoS One. 2021 Jul 6;16(7):e0254044. doi: 10.1371/journal.pone.0254044 (PMC8259970; doi:10.1371/journal.pone.0254044)
Supplement: Attachment — Submitted filename: PONE-D-20-38313_REPORT.pdf [file pone.0254044.s002.pdf]

PLOS ONE

A hybrid simulation model to study the impact of combined interventions on Ebola epidemic

--Manuscript Draft--

**Manuscript Number:**	PONE-D-20-38313
**Article Type:**	Research Article
**Full Title:**	A hybrid simulation model to study the impact of combined interventions on Ebola epidemic
**Short Title:**	study the impact of combined interventions on epidemic
**Corresponding Author:**	Peiyu Chen<br>Tsinghua University<br>Beijing, CHINA
**Keywords:**	hybrid method simulation; epidemic; health care works
**Abstract:**	Pandemics are recognized as a serious threat to human welfare all over the world. To effectively and efficiently reduce the mobility and mortality caused by the infectious disease, considerable models have been built to investigate the mechanism of the spread of infectious diseases and finding possible measures for preventing epidemic outbreaks in recent years. However, the commonly seen system dynamic epidemic models cannot easily integrate spatial and temporal heterogeneities. Nevertheless, agent-based epidemic models which have been widely used in a microscopic level have trouble modeling in the landscape where giant populations are at stake. To overcome these limits, we proposed a hierarchical hybrid modeling and simulation method which aims to study the dynamics of epidemic spreading. Through our method, we built a extended SEIR model, coupling system dynamics(SD) and Agent-based modeling(ABM), which considered not only the dynamics of sub-populations but also the individual heterogeneity. Besides, the essential characteristic of the impact of combined interventions on infectious disease epidemic was also revealed by our proposed model. Based on data from the 2014-2015 Ebola virus disease(EVD) in Sierra Leone, we parameterized and validated our proposed hybrid model and studied the effects of health care worker-targeted pre-deployment training and vaccination strategies. According to the simulation results, we identified an optimal combination of vaccine deployment and pre-job training strategies. To conclude, our hybrid model can be applied to help informing the synergistic disease control strategies and the corresponding hierarchical hybrid modeling and simulation method can further be used to reveal the effect of human behavior on epidemic spreading and examine the effectiveness of countermeasures against emerging and re-emerging infectious disease.
**Order of Authors:**	Peiyu Chen
	Wenhui Fan
	Xudong Guo
**Additional Information:**	
**Question**	**Response**
**Financial Disclosure**<br><br>Enter a financial disclosure statement that describes the sources of funding for the work included in this submission. Review the submission guidelines for detailed requirements. View published research articles from *PLOS ONE* for specific examples.	The author(s) received no specific funding for this work.

Powered by Editorial Manager® and ProduXion Manager® from Aries Systems Corporation

This statement is required for submission and **will appear in the published article** if the submission is accepted. Please make sure it is accurate.<br><br>**Unfunded studies**<br>Enter: *The author(s) received no specific funding for this work.*<br><br>**Funded studies**<br>Enter a statement with the following details:<br>• Initials of the authors who received each award<br>• Grant numbers awarded to each author<br>• The full name of each funder<br>• URL of each funder website<br>• Did the sponsors or funders play any role in the study design, data collection and analysis, decision to publish, or preparation of the manuscript?<br>• **NO** - Include this sentence at the end of your statement: *The funders had no role in study design, data collection and analysis, decision to publish, or preparation of the manuscript.*<br>• **YES** - Specify the role(s) played.<br><br>* typeset	
**Competing Interests**<br><br>Use the instructions below to enter a competing interest statement for this submission. On behalf of all authors, disclose any competing interests that could be perceived to bias this work—acknowledging all financial support and any other relevant financial or non-financial competing interests.<br><br>This statement **will appear in the published article** if the submission is accepted. Please make sure it is accurate. View published research articles from *PLOS ONE* for specific examples.	The authors have declared that no competing interests exist.

**NO authors have competing interests**<br><br>Enter: *The authors have declared that no competing interests exist.*<br><br>**Authors with competing interests**<br><br>Enter competing interest details beginning with this statement:<br><br>*I have read the journal's policy and the authors of this manuscript have the following competing interests: [insert competing interests here]*<br><br>* typeset	
**Ethics Statement**<br><br>Enter an ethics statement for this submission. This statement is required if the study involved:<br><br>• Human participants<br>• Human specimens or tissue<br>• Vertebrate animals or cephalopods<br>• Vertebrate embryos or tissues<br>• Field research<br><br>Write "N/A" if the submission does not require an ethics statement.<br><br>General guidance is provided below. Consult the submission guidelines for detailed instructions. **Make sure that all information entered here is included in the Methods section of the manuscript.**	N/A

Powered by Editorial Manager® and ProduXion Manager® from Aries Systems Corporation

**Format for specific study types**<br><br>**Human Subject Research (involving human participants and/or tissue)**<br>• Give the name of the institutional review board or ethics committee that approved the study<br>• Include the approval number and/or a statement indicating approval of this research<br>• Indicate the form of consent obtained (written/oral) or the reason that consent was not obtained (e.g. the data were analyzed anonymously)<br><br>**Animal Research (involving vertebrate animals, embryos or tissues)**<br>• Provide the name of the Institutional Animal Care and Use Committee (IACUC) or other relevant ethics board that reviewed the study protocol, and indicate whether they approved this research or granted a formal waiver of ethical approval<br>• Include an approval number if one was obtained<br>• If the study involved *non-human primates*, add *additional details* about animal welfare and steps taken to ameliorate suffering<br>• If anesthesia, euthanasia, or any kind of animal sacrifice is part of the study, include briefly which substances and/or methods were applied<br><br>**Field Research**<br><br>Include the following details if this study involves the collection of plant, animal, or other materials from a natural setting:<br>• Field permit number<br>• Name of the institution or relevant body that granted permission	
**Data Availability**<br><br>Authors are required to make all data underlying the findings described fully available, without restriction, and from the time of publication. PLOS allows rare exceptions to address legal and ethical concerns. See the PLOS Data Policy and FAQ for detailed information.	Yes - all data are fully available without restriction

Powered by Editorial Manager® and ProduXion Manager® from Aries Systems Corporation

A Data Availability Statement describing where the data can be found is required at submission. Your answers to this question constitute the Data Availability Statement and **will be published in the article**, if accepted.<br><br>**Important:** Stating 'data available on request from the author' is not sufficient. If your data are only available upon request, select 'No' for the first question and explain your exceptional situation in the text box.<br><br>Do the authors confirm that all data underlying the findings described in their manuscript are fully available without restriction?	
**Describe where the data may be found in full sentences. If you are copying our sample text, replace any instances of XXX with the appropriate details.**<br><br>• If the data are **held or will be held in a public repository**, include URLs, accession numbers or DOIs. If this information will only be available after acceptance, indicate this by ticking the box below. For example: *All XXX files are available from the XXX database (accession number(s) XXX, XXX.*).<br>• If the data are all contained **within the manuscript and/or Supporting Information files**, enter the following: *All relevant data are within the manuscript and its Supporting Information files.*<br>• If neither of these applies but you are able to provide **details of access elsewhere**, with or without limitations, please do so. For example:<br><br>*Data cannot be shared publicly because of [XXX]. Data are available from the XXX Institutional Data Access / Ethics Committee (contact via XXX) for researchers who meet the criteria for access to confidential data.*<br><br>*The data underlying the results presented in the study are available from (include the name of the third party*	All relevant data are within the manuscript and its Supporting Information files.

and contact information or URL).<br>• This text is appropriate if the data are owned by a third party and authors do not have permission to share the data.<br><br>* typeset	
Additional data availability information:	

Manuscript Click here to access/download;Manuscript;Manuscript.pdf

A hybrid simulation model to study the impact of combined interventions on Ebola epidemic

Peiyu Chen[1*], Wenhui Fan[1], Xudong Guo[1]

1 Department of Automation, Tsinghua University, Beijing, China

* cpy19@mails.tsinghua.edu.cn

Abstract

Pandemics are recognized as a serious threat to human welfare all over the world. To effectively and efficiently reduce the mobility and mortality caused by the infectious disease, considerable models have been built to investigate the mechanism of the spread of infectious diseases and finding possible measures for preventing epidemic outbreaks in recent years. However, the commonly seen system dynamic epidemic models cannot easily integrate spatial and temporal heterogeneities. Nevertheless, agent-based epidemic models which have been widely used in a microscopic level have trouble modeling in the landscape where giant populations are at stake. To overcome these limits, we proposed a hierarchical hybrid modeling and simulation method which aims to study the dynamics of epidemic spreading. Through our method, we built a extended SEIR model, coupling system dynamics(SD) and Agent-based modeling(ABM), which considered not only the dynamics of sub-populations but also the individual heterogeneity. Besides, the essential characteristic of the impact of combined interventions on infectious disease epidemic was also revealed by our proposed model. Based on data from the 2014-2015 Ebola virus disease(EVD) in Sierra Leone, we parameterized and validated our proposed hybrid model and studied the effects of health care worker-targeted pre-deployment training and vaccination strategies. According to the simulation results, we identified an optimal combination of vaccine deployment and pre-job training strategies. To conclude, our hybrid model can be applied to help informing the synergistic disease control strategies and the corresponding hierarchical hybrid modeling and simulation method can further be used to reveal the effect of human behavior on epidemic spreading and examine the effectiveness of countermeasures against emerging and re-emerging infectious disease.

Introduction

The rapid spread of an infectious disease can have a devastating impact on human welfare, lowering the quality of people's lives and causing increased mortality. This epidemic was officially declared a "public health emergency of international concern" by the World Health Organization(WHO) in 2014 [1, 2]. To effectively and efficiently reduce the epidemic size that cased by outbreaks of emerging infectious diseases, considerable efforts have been made for investigating the mechanism of the spread of infectious diseases and making informed decisions for controlling epidemic size.

Mathematical models have been a powerful tool to reveal the effect of human behavior on epidemic spreading and examine the influence of countermeasures against emerging and re-emerging infectious diseases [3–5]. The first model for the dynamics of

disease transmission in populations is the Susceptible-Infected-Recovered (SIR) model introduced by Kermack and McKendrick [6] which is based on system dynamics modelling(SDM). SDM describes the epidemic dynamics of sub-populations in spatially separated patches connected via transmission channels and deals with individual mobility at a macroscopic level [7]. This model was further developed by ~~Legrand~~ J et al. by proposing a stochastic SEIR model to fit data from the 1995 and 2000 outbreaks in Congo and Uganda [8]. Furthermore, Tsano et al. [9] studied the potential impact of environmental prophylactic vaccine on the dynamics of the infectious disease transmission. However, the stochastic nature of the movement of the individual is not considered explicitly with SDM for they only describe the mobility dynamics of sub-populations [10].

One the other hand, the agent-based modeling(ABM) has been widely used to simulate the dynamics of epidemics at a microscopic level which assumes stochastic processes of infection, recovery, birth, death, and movement events [11–13]. ABM can and has been used to examine how multi-level policies and programs influence population health [11]. However, it is tough to gain an insight of the epidemic dynamics with vole populations through ABM due to the massive calculations.

To prevent an infectious disease from spreading, various epidemic models have been designed to help public health agencies make effective decisions. Some researchers have focused on single-focus strategies [14–16] while other works analyzed combinations of intervention strategies [17–19]. [18] investigated how contact-reducing interventions and availability of antiviral drugs or vaccines contribute to the epidemic dynamics. [19] modeled and evaluated the spread of epidemic with intervention strategies of workforce shift and combination with school closure. In consideration of different intervention strategies, the spatial spreading of epidemics cannot be treated by deterministic compartmental models assuming a well-mixed population. However, such a model is yet to be established and this issue has motivated the present study.

The unprecedented outbreak of Ebola virus disease(EVD), a significant public health problem, in West Africa during 2014−2016 led to 28,638 cases, with 11,316 deaths as of 20 January 2016 [20]. The current EVD outbreak in West Africa is unprecedented in many ways, including the high number of doctors, nurses, and other health workers who have been infected. A new WHO report into health worker infections has found that health workers are between 21 and 32 times more likely to be infected with Ebola than people in the general population [20]. In many epidemics, Health care worker(HCW) has been the transmission link to the general population, acting as 'super-spreaders' in early epidemics. As they are a high-risk and relatively easily identified group, it is necessary to evaluate the impact of health care worker-targeted intervention strategies. Vaccination is known as one of the most effective and efficient intervention strategies for reducing the morbidity and mortality during the epidemic outbreak. Multiple mathematical modeling analyses aimed to evaluate the impact of vaccination during the outbreak of Ebola epidemics have been undertaken [21–24]. Besides, pre-deployment training for HCW also plays an important role in reducing infections. Studies indicate the low level of knowledge, negative attitude and sub-standard practices that can be eliminated through continued training of HCWs as well as provision of needed and adequate resources in their line of duties [25, 26]. However, few studies have incorporated both prophylactic vaccination strategies and the pre-deployment training strategies into the Ebola dynamic model.

In this paper, we proposed a new hierarchical hybrid modeling and simulation method, coupling SDM and ABM, which provides the advantages of each approach (spatial heterogeneity and individual dynamics for ABM and population size and analytical solution for SDM). Based on the characteristic of Ebola virus transmission in West Africa, we considered the problem of how to study the epidemic dynamics in vole

population while the individual heterogeneity is also considerable. We developed a hybrid model based on our hierarchical structure to simulate the dynamics of Ebola epidemic and to evaluate the impact of intervention strategies. Different vaccination and training strategies acting on HCW were incorporated into the proposed hybrid model to characterize the outbreak of Ebola so as to help making strategic decisions. The published data by WHO for 2014-2015 epidemic in Sierra Leone were used to validate our model and the recommended vaccination and training strategies were given for effectively preventing the spread of disease.

Our work made three contributions. Firstly, we proposed a hierarchical hybrid modeling and simulation method which constructed infectious disease transmission dynamics at different scales with the "Macroscopic" model (based on SDM) describing the general population on the size of a potential outbreak while "microscopic" model (based on ABM) describing how multi-level policies and programs shape population health. Secondly, we designed an extended SEIR epidemic model based on the newly raised hierarchical modeling method, which intended to take into account both vaccination and training strategies on HCW to better simulate the dynamics of Ebola epidemic and their treatment. Thirdly, we revealed the essential characteristic of the impact of combined interventions on infectious disease , extending the previous work by taking synergistic strategies into account.

The content of this paper is organized as follows. Section II describes the proposed hierarchical hybrid modeling and simulation method and the corresponding hybrid model relating to the dynamics of disease transmission during the 2014 EVD outbreak in Sierra Leone. Section III presents the validation results to evaluate the performance of our proposed model and studies the effects of combination of intervention strategies. Section IV illustrates our conclusion and discussion of the previous work as related to intervention strategies and the advantages of the proposed hybrid model comparing with existing epidemic simulation models as well as the future work based on our present study.

Methods

Hybrid modeling and simulation method

Traditional epidemiological models are based on the 'susceptible, exposed, infected, and removed (SEIR)' framework or its variants. However, considering the impact of intervention strategies in the emergency response, the disease transmission dynamic model needs to have spatial heterogeneity. So we proposed a hybrid modeling and simulation method using ABM in the micro level and SDM in the macro level. In our method, epidemiological models based on 'SEIR' framework were used at the macro level, inside which agents were modeled at the micro level (ABM part). The mobility dynamic of sub-populations was described on the macro level while the individual heterogeneity was shown in the micro level. An illustration of the ABM method based on SD in the hybrid model is presented in fig 1.

Fig 1. Hybrid modeling and simulation method The rectangular in macro level represents the sub-population and the icons in micro level represent the agents.

Model data

The epidemic of Ebola in Sierra Leone was simulated and validated using specific disease characteristic parameters derived from literature researches and

epidemic-specific parameters estimated by simultaneously minimizing the squared differences between the model output of infectious cases in the overall population and the cases reported by WHO for the 2014 epidemic and the squared differences between the model output of HCW cases and the cases as reported [27, 28]. The simulation period (from May 12, 2014 to November 13, 2015) is divided into three phases (first period: May 12, 2014 to September 12,2014 a total of 123 days, second period: September 12,2014 to November 18,2014, a total of 67 days, third period: November 19,2014 to November 13, 2015, a total of 360 days) according to their population dynamics characteristic. The reported data is shown in fig 2.

Fig 2. Reported Ebola cases for 2014-2015 Sierra Leone epidemic among overall population and among HCWs. Data for the overall population cases were reported by WHO [27] and Data for HCW cases were collected by Fang et al. [28]. A: Reported incidence in the overall population in 2014-2015 Sierra Leone epidemic(cumulative cases). B: Weekly Ebola cases among HCWs(weekly cases).

Model assumption

The main purpose of this model is to simulate the outbreak of Ebola and facilitate analysis of different education and vaccination scenarios. Considering the real affection of vaccination and education, we made the following assumptions:

1. The birth rate and death rate of the total population in Sierra Leone and the increase of HCW do not take into account in the model.
2. Vaccine assumed: time to onset of protection: 7 days. Duration of protection: 180 days. Efficacy: 100%. Daily rate at which vaccination is carried out: 5% HCW.
3. Only trained HCW have opportunities to be vaccinated.
4. There are several phases in the education and the probability of being infected correlates with the level of education.
5. Education assumed: HCWs who were willing to seek training can get pre-deployment training.

Model

Based on the case of Ebola epidemic in Sierra Leone, an epidemic model using the hybrid modeling and simulation method was built in order to study the effect of the policy interventions (vaccination and education) working on HCW and to formulate recommendations for potential future health-related emergency responses for the government.

System dynamics modeling

According to [21], the study expended the general SEIR model which facilitate differentiation between HCW and the general population. In our study, we introduced a related and also new model with a difference. In the SDM, we used a similar structure as [21]. The population in Sierra Leone was divided into and moved between several infection states overtime and each state was assumed to be a collection of homogeneous population. In addition to the general SEIR model, the sub-population "susceptible(S)" were seperated into "susceptible HCW" and "susceptible general population" as shown in fig 3.

Fig 3. System dynamics modeling in the hybrid model. S_HCW means susceptible HCW and S_G means susceptible general population. The "susceptible HCW" sub-population acted as linkage between the agent-based modeling and the system dynamics modeling

Briefly, Susceptible individuals (S) may become Exposed (E) after contact with an infectious individual and transform into the Infectious (I) class after the virus incubation period, thereafter be capable of infecting others. A proportion of these individuals may be Hospitalized (H). Both untreated patients in I and hospitalized patients in H may lead to one of two outcomes: they may be dead(D), with a chance of infecting others before being buried or having a funeral (F), or they may recover(R). The transmissions and the ordinary differential equations describing this model are listed in the supplemental material S1 File.Supporting data.

Agent-based modeling

In this study, we assumed HCW as "agent" to analyze their behavior within a given strategy. With the help of ABM, HCW can be simulated in a micro level where the independent and synergistic effects of multiple interventions on the HCW can be modeled. The micro level model structure is shown in Fig4,5 and 6. Considering the vaccination and education strategies, our micro level model was separated into two modules: education and vaccination. Each HCW changes his or her state according to the rules of transition ().

Fig 4. The education module in the agent-based modeling. The state chart presents each HCW's behavior after the onset of EVD outbreak to the point of being well trained or lack of training in which the rectangular boxes represent the states of HCWs and the diamond means branch from which the solid arrow represents transition under a certain condition (ex. "Yes"), and the dashed arrow represents the default (ex. "No").

In the education module, HCWs were separated into several types according to their academic certificate and their willingness to work. The arrows in Fig4 represent the transitions according to the rules presented in the earlier study [29]. We mainly focused on the pre-deployment training which prepared for HCW in the Ebola affected area. Other factors which represent the situation of education were also considered in the ABM. We specifically designated the parameters that are related to the amount of schooling (professional training for HCWs) as several decision making processes (diamond shapes in Fig4) and their corresponding transition rate P1-P3. The meaning and value of these three parameters are are described in Table2. Here, we focused on the rate of P3 which explains the coverage of pre-deployment training among HCWs.

At the first stage of the state chart(Fig4), those who had knowledge of EVD before the outbreak turned into "Initial Ebola Education" state while others turned into the state of "Novice". At the second stage of the state chart, those who had knowledge of EVD before the outbreak attended intensive training according to the transmission rate P2 while those who have low level of knowledge attend the pre-deployment training according to the transmission rate P3. After these two stages, those who did not attend the training were "unprotected" and eventually became "Exposed" through the time transition of 2 to 21 days (the incubation period of the Ebola virus) when carrying out duties in health facilities. Those who attended the training moved to the "trained" state and thus carried out appropriate infection, prevention and took the initiative to get vaccinated.

Fig 5. The vaccination module in the agent-based modeling. ”V1” represents HCWs who have already vaccinated but the efficacy of vaccine has not been onset yet. ”V2” means HCWs who were protected by the effective vaccine.

Fig 6. The structure of the model of micro level based on the activity of HCW. The Education module and the vaccination module were concentrated by the state ”Trained” which was both described in the two modules. The arrows demonstrate the available state transition path.

HCWs who had attended intensive training (HCWs who were in the “Trained” state) moved into the vaccination module(Fig5) according to the vaccine penetration. The two modules were connected though the state of ”trained” as shown in Fig6. Considering the vaccine characteristic which includes the onset of protection and the duration of protection, agents in this phase were separated into several states: i. HCWs who have already vaccinated but the efficacy of vaccine has not been onset yet (V1), ii. HCWS who were protected by the effective vaccine (V2), iii. Vaccinated HCWs reverted to the susceptible state depending on expiration of vaccine efficacy (remain in the state “Trained”). The transformation rule of these three states followed the vaccine characteristics. The transition paths are listed as followed:

1. HCWs in “trained” state moved to “V1” according to the vaccine penetration.
2. HCWs in “trained” state turned to be “Exposed” before getting vaccination.
3. HCWs in “V1” state turned to be “Exposed” before the onset of the efficacy of vaccine.
4. HCWs in “V1” state moved to “V2” as the vaccine starts taking effect.
5. HCWs in “V2” state reverted to “Trained” state upon expiration of vaccine efficacy.

Experimental design

In order to analyze the effects of implementation of both the independent and synergistic interventions, both the scene under independent intervention and synergistic interventions need to be considered.

1. Without interventions: The simulation ran with the parameters that are parameterized and validated by the data reported by WHO as a comparison.
2. Pre-deployment training strategies: The impact of education strategies was assessed by raising the rate of P3 from 95% to 100% at a step of 1%. Additionally, the value of P3 was 95% in the scenario that without interventions.
3. Vaccination strategies: The strategies were evaluated by prophylactic vaccination of 10%, 30% and 50% of all HCWs compared with no-vaccination scenario.
4. Combined intervention strategies: The rate of P3 was varied from 95% to 100% at a step of 1% with 10% ,30% or 50% HCWs got vaccination.

The number of cases and deaths in each scenario and differences in such cases and deaths between any two scenarios being compared were used to represent the size of the epidemic and the effect of interventions.

Results

Model parameterization and validation

The parameters fitted to the published data by WHO for 2014-2015 epidemic in Sierra Leone can be seen in Table 1 and Table 2. The comparison between the model output of Ebola cases and deaths among total population and among HCWs in Sierra Leone with the relative data reported by WHO are shown in Fig7. The deterministic model fit well for both the cases in the overall population and the cases among HCWs, with the predicted curve of cumulative cases following the reported number of cases in both groups. The starting date of the x-coordinate "day 0" represents March 22,2014 while the end of the simulation date represents November 13,2015.

Fig 7. Comparison of the output of the base case model with published data. A: Comparison of cumulative Ebola cases reported by the WHO with model output. B: Comparison of cumulative Ebola mortality reported by the WHO with model output. C: Comparison of cumulative HCW Ebola cases reported by Fang et al. with model output.

The mean model outputs showed 14456 cases and 4048 deaths compared to 14122 cases and 3955 deaths reported by WHO. Considering the group of HCWs, the mean model outputs showed 312 cases compared to 299 cases reported by WHO. The basic reproduction number R0 for this hybrid model is 1.30($1.26 \leq R0 \leq 2.53$) which is consistent to the reported estimation of R0 for the 2014 Sierra Leone epidemic in the earlier work [36].

Impact of pre-deployment training strategies

To evaluate the impact of pre-deployment training, Fig8 shows the simulated curve for the dynamic of the Ebola epidemic respect to the accumulated infected population under different coverage rate of pre-deployment training among HCW and under the scenario with an initial case (without interventions). Additionally, although the value of P3 in the initial case may not precisely reveal the real situation, the bias of P3 has little impact on our study for we mainly focused on the trend rather than the fluctuation of the value. It can be seen that i. a small increase of the number of HCW (95% to 96% HCW) getting pre-deployment training can help reduce both cases and deaths at the overall population level by 31.85% (Table 3). ii. About 10% of Ebola cases and deaths among general population can be reduced with 1% increase in the coverage of pre-deployment training for HCW. iii. Enhancing the education for the frontline HCW can have a positive impact on the disease prevention.

Fig 8. Impact of pre-deployment training of different proportions of HCW on cumulative Ebola cases and mortality. A: Cumulative cases. B: Cumulative deaths.

Impact of vaccination strategies

To evaluate the impact of vaccination, we compared the simulation results between the epidemic in the event of prophylactic vaccination of different proportions of HCW and that of the original cases where no preventive vaccination was carried out (Fig9). The model output showed that with 10% HCW getting vaccination (about 115 HCWs which make up 0.0016% of the population in Sierra Leone), 54% of cases and 51% of deaths at

Table 1. Parameters for the hybrid model

Parameter	Description	0–123 Days	123–190 Days	190–550 Days	Source
N	Size of the total population in Sierra Leone (2014)	7,017,144			[30]
HCW	Health care worker	1153			[31]
$1/\sigma$	Incubation Period	7 days	7 days	7 days	[8]
$1/\gamma_D$	Mean duration from death to burial	2 days	2 days	2 days	[8]
$1/\alpha$	Mean duration from onset of infection to hospitalization	2.4 days	2.4 days	2.2 days	[21]
β_{IHCW}	Contact rate for infectious individuals in HCW	117.8	15	5.1	
β_{HHCW}	Contact rate for hospitalized individuals in HCW	189.21	23.64	8.88	
β_{DHCW}	Contact rate for dead but not buried individuals in HCW	0.0726	0.052	0.045	
β_{IHCW}	Contact rate for infectious individuals in general population	0.635	0.594	0.425	
β_{HNHCW}	Contact rate for hospitalized individuals in general population	0.002	0.001	0.0008	
β_{DNHCW}	Contact rate for dead but not buried individuals in general population	0.07	0.0514	0.048	
$\delta 1$	Case fatality rate for unhospitalized individuals	0.46	0.4	0.2	
$\delta 2$	Case fatality rate for hospitalized individuals	0.46	0.4	0.2	
$1/\gamma$	Mean duration from onset of infection to death or recovery	6 days	6 days	6 days	[21]
$1/\gamma_H$	Mean duration from hospitalization to death or recovery	6.2 days	8.3 days	16 days	[21]
K1	Proportion of HCW among the total population at the start of the epidemic	0.016%			Calculated

Parameters that are not tagged with sources were fitted to the published data according to the basic model output.

Table 2. Parameters for the hybrid model(education related)

Parameter	Description	Value	Source
P	The number of HCWs trained in a week	100 HCWs	[32]
T	Duration of intensive training	5 days	[33].
NK	proportion of HCW who stopped working did not have knowledge of EVD	60%	[33]
P1	the percentage of the total HCWs that had knowledge of EVD before the outbreak	10	[32, 34, 35]
P2	the percentage of HCWs who had knowledge of EVD before the outbreak and attended intensive training during the outbreak	90%	[32, 34, 35]
P3	the probability for unprepared and unskilled frontline HCW to attend pre-deployment training during the outbreak	95%	[32, 34, 35]

Table 3. Impact of pre-deplyment training for healthcare workers on cumulative cases and mortality among overall population caused by EVD.

Parameter	95% of HCW	96% of HCW	97% of HCW	98% of HCW	99% of HCW	100% of HCW
Number trained	1095	1107	1118	1130	1141	1153
Cumulative cases	14456	9841	8668	7363	5380	4206
Proportion of cases averted vs no intervention	-	31.92%	40.04%	49.07%	62.78%	70.90%
Cumulative deaths	4048	2760	2402	2038	1481	1168
Proportion of deaths averted vs no intervention	-	31.82%	40.66%	49.65%	63.41%	71.15%

the whole population can be ~~avoid~~ and the efficiency of vaccination can be described that 67.37 cases can be averted by per vaccination. In the scenario that vaccination of 30% HCW, the efficiency of vaccination can be described as 26.18 cases can be averted by per vaccination. Also, the vaccination of 50% HCW helped reduce 16.39 cases per vaccination (Table 4). The efficiency of vaccination can be referred to estimate the time for carrying out vaccinations among certain group. However the vaccination time was also influenced by the infrastructural challenges, the time the vaccination taken for onset of protection and also the degree of dispersion of the people who need vaccination. The simulation result indicated that i. vaccination of a small proportion of populations can effectively reduce the size of epidemic compared with the study result that effective EVD vaccination rates were estimated to be 42% for the overall population [37] for the reason that vaccinating HCWs can be seen as an effective method to eliminate virus transmission channels between HCW and the infected people. ii. The proportion of cases averted ~~comparing~~ to the results of no vaccination scenario corresponding to the vaccination of 30% and 50% HCW are similar. This result indicated that the effects of vaccination on HCW are limited. iii. Considering the efficiency of vaccination, vaccination of 30% HCW is recommended.

Fig 9. Impact of prophylactic vaccination of different proportions of HCW on cumulative Ebola cases and mortality. A: Cumulative cases. B: Cumulative deaths.

Table 4. Impact of vaccination for healthcare workers on cumulative cases and mortality among overall population caused by EVD

**Parameter**	**No vaccination**	**10% of HCW vaccinated**	**30% of HCW vaccinated**	**50% of HCW vaccinated**
Number vaccinated	0	115	345	575
vaccination efficiency/per vaccination	-	67.37	26.18	16.39
Cumulative cases	14456	6709	5425	5033
Proportion of cases averted vs no vaccination	-	53.59%	62.47%	65.18%
Cumulative deaths	4048	1987	1611	1492
Proportion of deaths averted vs no vaccination	-	50.91%	60.20%	63.14%

Impact of combination of vaccination and education strategies

To explore the effects of the combined intervention strategies, we varied the pre-deployment training rate from 95% to 100% at a step of 1% while the values of the vaccination coverage varied from 10% to 50% with increments of 20%. The simulation results of the hybrid model which collected the epidemic cases and deaths of scenarios with respect to different intervention combinations is demonstrated in Fig10. Fig11 depict the median, interquartile range and 1.5 times the interquartile range of the simulation output under different intervention strategies. Fig11 showed the median decrease in Ebola cases and deaths as compared to the baseline(result of the scenario without intervention) combining intensified training and an increase of vaccination proportion. To further investigate the effects of the segregation strategies, Fig12

illustrated the simulation results by the aversion rate of cumulative incidence and mortality compared with the no intervention scenario.

Fig 10. Impact of combined interventions on cumulative Ebola cases and mortality. The combined interventions refers to combination of education and vaccination strategies. A: Cumulative cases. B: Cumulative deaths.

Fig 11. The median, interquartile range and 1.5 times the interquartile range of the simulation output under different combination of intervention strategies. A-D: Cumulative cases under different combination of intervention strategies. E-H: Cumulative deaths under different combination of intervention strategies.

Fig 12. Impact of combined interventions on percentage of cases or deaths averted related to no vaccination scenario. A: Cases averted related to no vaccination scenario. E-H: Deaths averted related to no vaccination scenario.

Respectively, Table 5 and 6 shows the cases, deaths as well as the proportion of cases averted versus the cases in no intervention scenarios corresponding to the result in Fig10 and 11. It can be observed that the size of the accumulated infected population decreases gradually with the increase of education rate under the same coverage proportion of vaccination, as is shown in Fig10 and 11. However, under the same education rate, the degree of influence of different vaccination strategies under the synergistic interventions showed difference comparing to the single intervention cases as the cumulative incidence and mortality do not keep a remarkable decrease with the increase of vaccination rate. According to Table 5, the proportion of cases averted were seen to be 31.92% without vaccination as compared to 67.26% with prophylactic vaccination of 10% of HCW with P3 kept the rate of 0.96. 35.34% cases averted by the intervention of increasing 10% HCW to get vaccination.

In contrast, vaccination of 50% of HCW with 96% HCW getting trained resulted in an aversion of 70.52% cases which means that only 3.26% cases can be averted by the intervention of increasing 40% HCW to get vaccination. Comparing with the scenarios that without education intervention strategies, 53.59% of cases can be averted when 10% of HCW get vaccination while 62.47% of cases be averted with 30% vaccination rate. A 8.88% increase was seen between the '10% vaccination' strategy and '30% vaccination' strategy. With the further increase of the coverage of pre-deployment training, the downward shift in the overall Ebola cases and deaths become less pronounced among the vaccination rate of 10%, 30% and 50%.

Based on Fig12, Table 5 and 6, there are several conclusions to be made concerning these results. i. The combination of interventions for Ebola prevention and protection was more effective than the individual intervention. ii. Although the combination of interventions was seen to add substantial benefit to the epidemic prevention and protection, the efficiency of expanding vaccination coverage showed a decline when the education strategies are implement simultaneously. iii. Unlike the recommended strategies given in the 'Impact of vaccination strategies', vaccination of 10% of HCW can achieve a higher efficiency than vaccination of 30% of HCW. In other words, with the intervention of expanding the coverage of pre-deployment training for HCW, 10% of vaccination rate is recommended in the process of Ebola epidemic prevention and protection.

Table 5. Impact of combined interventions on cumulative Ebola cases and the proportion of cases averted versus no vaccination scenarios.

Vaccination rate	P3					
	0.95	0.96	0.97	0.98	0.99	1
0	14456	9841	8668	7363	5380	4206
Proportion of cases averted vs no vaccination	0	31.92%	40.04%	49.07%	62.78%	70.90%
0.1	5478	4733	3771	2839	2035	1135
Proportion of cases averted vs no vaccination	62.11%	67.26%	73.91%	80.36%	85.92%	92.15%
0.3	5293	4400	3346	2624	2047	1203
Proportion of cases averted vs no vaccination	63.39%	69.56%	76.85%	81.85%	85.84%	91.68%
0.5	5012	4261	3770	2628	1970	1347
Proportion of cases averted vs no vaccination	65.33%	70.52%	73.92%	81.82%	86.37%	90.68%

Table 6. Impact of combined interventions on cumulative Ebola deaths and the proportion of deaths averted versus no vaccination scenarios.

Vaccination rate	P3					
	0.95	0.96	0.97	0.98	0.99	1
0	4048	2760	2402	2038	1481	1168
Proportion of deaths averted vs no vaccination	0	31.82%	40.66%	49.65%	63.41%	71.15%
0.1	1623	1402	1118	841	603	336
Proportion of deaths averted vs no vaccination	59.91%	65.37%	72.38%	79.22%	85.10%	91.70%
0.3	1570	1312	992	777	607	353
Proportion of deaths averted vs no vaccination	61.22%	67.59%	75.49%	80.81%	85.00%	91.28%
0.5	1486	1263	1019	779	585	333
Proportion of deaths averted vs no vaccination	63.29%	68.80%	74.83%	80.76%	85.55%	91.77%

Discussion and Conclusion

Given that analysis mentioned above, we drew the following conclusions: i. The hybrid modeling and simulation method we proposed in this work can be used to construct the hybrid simulation method model based on the specific epidemic case. ii. The hybrid simulation model constructed in our study was fit to the 2014-2015 Ebola epidemic outbreak in Sierra Leone. iii. Our proposed hybrid model can simulate the implementation of synergistic interventions which can effectively slow down the epidemic. iv. Vaccination of 10% HCW in addition to intensified training on HCW during the outbreak could have a significant impact while further increasing the vaccination rate resulted in little improvement.

Our model combined mathematical equations for the transmission of subpopulation in epidemic affected area and multi-agent systems for the management of healthcare workers. Such mutiscale modeling method was also used in megapopulation dynamics modeling [38]. In order to evaluate the synergistic interventions on HCW, our hybrid model used ABM to simulate HCW's reactions to the interventions and SDM to simulate the dynamic of epidemic in the overall population. Additionally, the number of infections in HCW played the role of linking between the two modeling level. This model outlined a promising method to model large-scale systems by engaging various dynamics at different scales. The combination of SDM and ABM has many advantages: i. Easing dynamic representation by using different types of data and modeling at different scales (e.g., spatial scales and temporal scales). [39] ii. Modeling individual heterogeneity, rare events and processes occurring at various scale with vole populations.

Compared with the earlier studies of modelling the epidemic dynamics, our model overcomes the following difficulties: i. Gain an insight into the epidemic dynamics ,that has been studied by the traditional SEIR framework-based models or its variants based on SDM [40–42], in the real landscape where the individual heterogeneity, rare events, sophisticated population structure and the processes occurring at different scales must be altogether take into account. [38] ii. The computational difficulty of modeling vole population (7017144 people were taken into account in our study) using ABM.

The hybrid model proposed in our study provided a reliable method that helps to analyze the impact of healthcare related policies and give efficient combination strategies aiming to protect the high-risk populations against Ebola virus. Comparing our modeling result with other epidemic model which analyzing the intervention policies implemented for disease control and prevention, our work illustrated new findings in the presence of consistent conclusions with the earlier works. Ebola cases reported from Sierra Leone in HCWs represented a much higher estimated cumulative incidence of confirmed Ebola in HCWs than in non-HCWs [43] which lead to a high efficiency of HCW-targeted vaccination [44]. Robert A et al. used a mathematical transmission model to explore the relative contribution of HCW and the community to transmission and gave the conclusion that ahead-of-time HCW-targeted strategies had an impact at coverage of 30% [22] which is consistent to our conclusion given for the case that only vaccination strategies are considered. Besides, we also took into account the intervention of education and lead to the conclusion that HCW-targeted strategies had an impact at coverage of 10% on condition that pre-job training strategies are also considered, corresponding to the statement that the achievement of one policy objective may preclude success with others [45]. Alexis Robert's et al. estimated that prophylactic vaccination of 10% of HCW will result in 7685 cases and 3155 deaths while vaccination of 30% of HCW will result in 5776 cases and 2358 [21] compared with our model output 6709 cases and 1987 deaths with 10% vaccination rate and 5425 cases and 1611 deaths with 30% vaccination rate. We did not differentiate between confirmed, probable and suspected cases and regarded them as cases in our model owing to the structure of 'SEIR' model which does not explicitly distinguish these three type of cases. However, Alexis Robert's model only considered the confirmed cases which lead to differences in results. Our model can be seen as an extension or a calibration for the earlier study.

We have identified several improvements that can be made which will become the focus of future work. Firstly, the infections in HCW and the infections in general population were assumed to be homogeneous in our study. In the real world, this assumption cannot hold fully which may lead to a bias between the model output and the real data. Secondly, the relation between vaccination policy and education policy is much more sophisticated in the real world than that in our model.

To conclude, our hybrid simulation model successfully presented the dynamics of EVD transmission and evaluated the impact of different intervention strategies. Our hybrid modeling and simulation method which is a first step to develop a more specific understanding of epidemic spreading in vole population models with heterogeneous human mobility can help inform disease control strategies for different infectious disease.

Supporting information

S1 File.Supporting data (PDF)

Acknowledgments

The authors would like to thank the financial support given by The National Key Research and Development Program of China—Collective intelligence network simulation and the experimental platform development (2017YFB1400105).

Author Contributions

Conceived and designed the experiments : PC, WF. Performed the experiments: PC, XG. Collect and analyzed the data: PC, XG. Contributed reagents/materials/analysis: PC, WF. Writing-original draft: PC, WF. Writing-review and editing: PC, WF, XG.

References

1. Dixon MG, Schafer IJ. Ebola viral disease outbreak—West Africa, 2014. MMWR Morbidity and mortality weekly report. 2014;63(25):548.

2. World Health Organization. WHO statement on the 1st meeting of the IHR Emergency Committee on the 2014 Ebola outbreak in West Africa Geneva, Switzerland: World Health Organization;. `https://www.who.int/mediacentre/news/statements/2014/ebola-20140808/en/` cited 2017 November 30, 2017.

3. Nguyen C, Carlson JM. Optimizing Real-Time Vaccine Allocation in a Stochastic SIR Model. Plos One. 2016;11(4):e0152950.

4. Abbasi Z, Zamani I, Mehra AHA, Shafieirad M, Ibeas A. Optimal Control Design of Impulsive SQEIAR Epidemic Models with Application to COVID-19. Chaos Solitons & Fractals. 2020;139:110054.

5. Zhang Z. The outbreak pattern of SARS cases in China as revealed by a mathematical model. Ecological Modelling. 2007;204(3-4):420–426.

6. Kermack WO, McKendrick AG. A contribution to the mathematical theory of epidemics. Proceedings of the royal society of london Series A, Containing papers of a mathematical and physical character. 1927;115(772):700–721.

7. Forrester JW. System dynamics, systems thinking, and soft OR. System dynamics review. 1994;10(2-3):245–256.

8. Legrand J, Grais RF, Boelle PY, Valleron AJ, Flahault A. Understanding the dynamics of Ebola epidemics. Epidemiology & Infection. 2007;135(4):610–621.

9. Tsanou B, Bowong S, Lubuma J, Mbang J. Assessing the impact of the environmental contamination on the transmission of Ebola virus disease (EVD). Journal of Applied Mathematics and Computing. 2017;55(1-2):205–243.

10. Vincenot CE, Giannino F, Rietkerk M, Moriya K, Mazzoleni S. Theoretical considerations on the combined use of System Dynamics and individual-based modeling in ecology. Ecological Modelling. 2011;222(1):210–218.

11. Urabe CT, Tanaka G, Aihara K, Mimura M. Parameter scaling for epidemic size in a spatial epidemic model with mobile individuals. PloS one. 2016;11(12):e0168127.

12. Tracy M, Cerdá M, Keyes KM. Agent-Based Modeling in Public Health: Current Applications and Future Directions. Annual Review of Public Health. 2018;39(1):annurev–publhealth–040617–014317.

13. El-Sayed AM, Scarborough P, Seemann L, Galea S. Social network analysis and agent-based modeling in social epidemiology. Epidemiologic Perspectives & Innovations. 2012;9(1):1–1.

14. Oleś K, Gudowska-Nowak E, Kleczkowski A. Understanding disease control: influence of epidemiological and economic factors. PloS one. 2012;7(5):e36026.

15. Jackson C, Mangtani P, Hawker J, Olowokure B, Vynnycky E. The effects of school closures on influenza outbreaks and pandemics: systematic review of simulation studies. PloS one. 2014;9(5):e97297.

16. Yuan EC, Alderson DL, Stromberg S, Carlson JM. Optimal vaccination in a stochastic epidemic model of two non-interacting populations. PLoS One. 2015;10(2):e0115826.

17. Grijalva CG, Goeyvaerts N, Verastegui H, Edwards KM, Gil AI, Lanata CF, et al. A household-based study of contact networks relevant for the spread of infectious diseases in the highlands of Peru. PloS one. 2015;10(3):e0118457.

18. Hollingsworth TD, Klinkenberg D, Heesterbeek H, Anderson RM. Mitigation strategies for pandemic influenza A: balancing conflicting policy objectives. PLoS Comput Biol. 2011;7(2):e1001076.

19. Zhang T, Fu X, Ma S, Xiao G, Wong L, Kwoh CK, et al. Evaluating temporal factors in combined interventions of workforce shift and school closure for mitigating the spread of influenza. PLoS One. 2012;7(3):e32203.

20. World Health Organization. Ebola in the Democratic Republic of the Congo: Health Emergency Update Geneva, Switzerland: World Health Organization; 2019;. https://www.who.int/emergencies/diseases/ebola/drc-2019.

21. Potluri R, Kumar A, Maheshwari V, Smith C, Oriol Mathieu V, Luhn K, et al. Impact of prophylactic vaccination strategies on Ebola virus transmission: A modeling analysis. Plos one. 2020;15(4):e0230406.

22. Robert A, Camacho A, Edmunds WJ, Baguelin M, Tamfum JJM, Rosello A, et al. Control of Ebola virus disease outbreaks: Comparison of health care worker-targeted and community vaccination strategies. Epidemics. 2019;27:106–114.

23. Bodine EN, Cook C, Shorten M. The potential impact of a prophylactic vaccine for Ebola in Sierra Leone. Mathematical Biosciences & Engineering. 2018;15(2):337.

24. Xie Z. Data fitting and scenario analysis of vaccination in the 2014 Ebola outbreak in Liberia. Osong public health and research perspectives. 2019;10(3):187.

25. Olowookere SA, Abioye-Kuteyi EA, Adepoju OK, Esan OT, Adeolu TM, Adeoye TK, et al. Knowledge, attitude, and practice of health workers in a tertiary hospital in Ile-Ife, Nigeria, towards Ebola viral disease. Journal of tropical medicine. 2015;2015.

26. Tour&e A, Traor&e F, Sako F, Delamou A, Tonguino F, Sylla D, et al. Knowledge, attitudes, and practices of health care workers on Ebola virus disease in Conakry, Guinea: a cross-sectional study. Journal of Public Health and Epidemiology. 2016;8(2):12–16.

27. World Health Organization. Ebola situation reports: archive;. `http://who.int/csr/disease/ebola/situation-reports/archive/en/`.

28. Fang LQ, Yang Y, Jiang JF, Yao HW, Kargbo D, Li XL, et al. Transmission dynamics of Ebola virus disease and intervention effectiveness in Sierra Leone. Proceedings of the National Academy of Sciences. 2016;113(16):4488–4493.

29. Jones-Konneh TEC, Suda T, Sasaki H, Egawa S. Agent-Based Modeling and Simulation of Nosocomial Infection among Healthcare Workers during Ebola Virus Disease Outbreak in Sierra Leone. The Tohoku journal of experimental medicine. 2018;245(4):231–238.

30. The World Bank. Population, total in Sierra Leone (2014);. `https://data.worldbank.org/indicator/SP.POP.TOTL`.

31. Evans DK, Goldstein M, Popova A. The next wave of deaths from Ebola? the impact of health care worker mortality. The World Bank; 2015.

32. Macmillan P. International Organization for Migration (IOM); 2016.

33. Jones-Konneh TEC, Murakami A, Sasaki H, Egawa S. Intensive Education of Health Care Workers Improves the Outcome of Ebola Virus Disease: Lessons Learned from the 2014 Outbreak in Sierra Leone. The Tohoku Journal of Experimental Medicine. 2017;243(2):101–105.

34. Raven J, Wurie H, Witter S. Health workers' experiences of coping with the Ebola epidemic in Sierra Leone's health system: a qualitative study. Bmc Health Services Research. 2018;18(1):251.

35. Annan AA, Yar DD, Owusu M, Biney EA, Forson PK, Okyere PB, et al. Health care workers indicate ill preparedness for Ebola Virus Disease outbreak in Ashanti Region of Ghana. BMC Public Health. 2017;17(1):546.

36. Bodine EN, Cook C, Shorten M. The potential impact of a prophylactic vaccine for Ebola in Sierra Leone. Mathematical Biosciences & Engineering. 2018;15(2):337.

37. Guo Z, Xiao D, Li D, Wang X, Wang Z. Predicting and Evaluating the Epidemic Trend of Ebola Virus Disease in the 2014-2015 Outbreak and the Effects of Intervention Measures. Plos One. 2016;11(4):e0152438.

38. Vincenot CE, Mazzoleni S, Moriya K, Carteni F, Giannino F. How spatial resource distribution and memory impact foraging success: A hybrid model and mechanistic index. Ecological Complexity. 2015;22(jun.):139–151.

39. Nicolas M, Christophe L, Patrick G. Coupling agent-based with equation-based models to study spatially explicit megapopulation dynamics. Ecological Modelling. 2018;384:34–42.

40. Asher, Jason. Forecasting Ebola with a Regression Transmission Model. Epidemics. 2017; p. S175543651730021X.

41. A AD, B ABG. Modeling the impact of quarantine during an outbreak of Ebola virus disease. Infectious Disease Modelling. 2019;4:12–27.

42. Xie Z. Data Fitting and Scenario Analysis of Vaccination in the 2014 Ebola Outbreak in Liberia. Osong Public Health and Research Perspectives. 2019;10(3).

43. Kilmarx PH, Clarke KR, Dietz PM, Hamel MJ, Jambai A. Ebola virus disease in health care workers–Sierra Leone, 2014. Mmwr Morbidity & Mortality Weekly Report. 2014;63(49):1168.

44. Bodine EN, Cook C, Shorten M. The potential impact of a prophylactic vaccine for Ebola in Sierra Leone. Mathematical Bioences and Engineering. 2018;15(2):337–359.

45. Hollingsworth TD, Klinkenberg D, Heesterbeek H, Anderson RM. Mitigation Strategies for Pandemic Influenza A: Balancing Conflicting Policy Objectives. Plos Computational Biology. 2011;7(2):e1001076.

Click here to access/download;Figure;fig8.tif

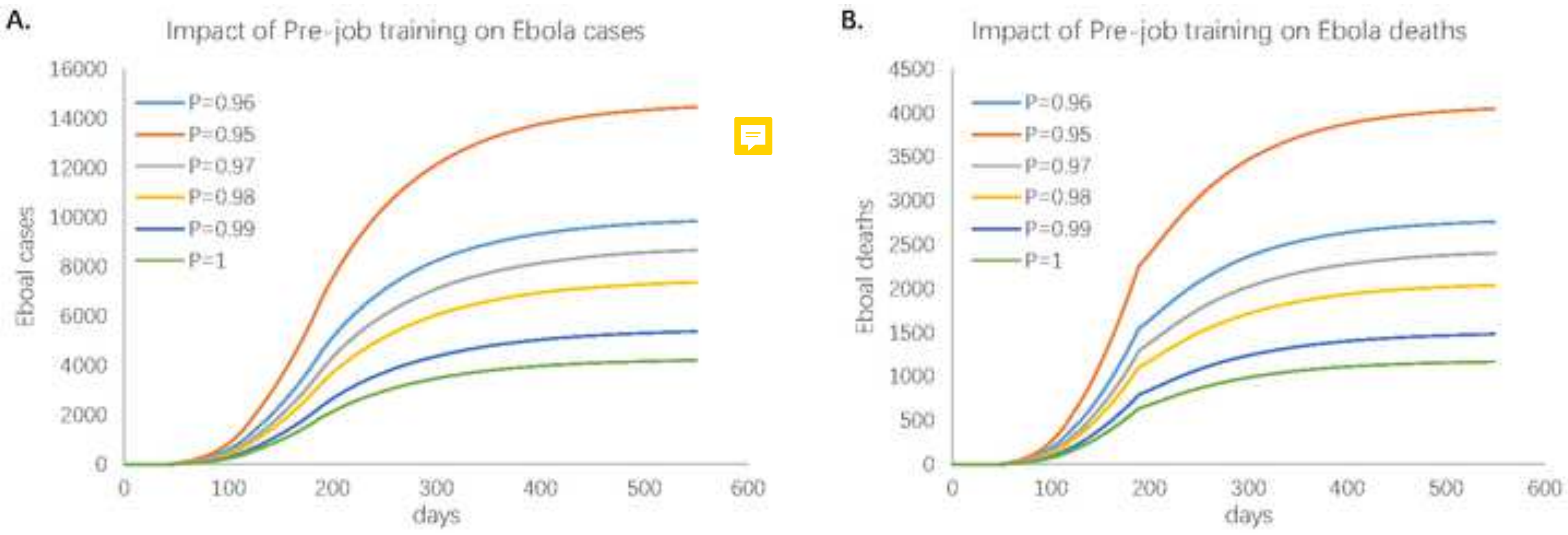

Click here to access/download;Figure;fig9.tif

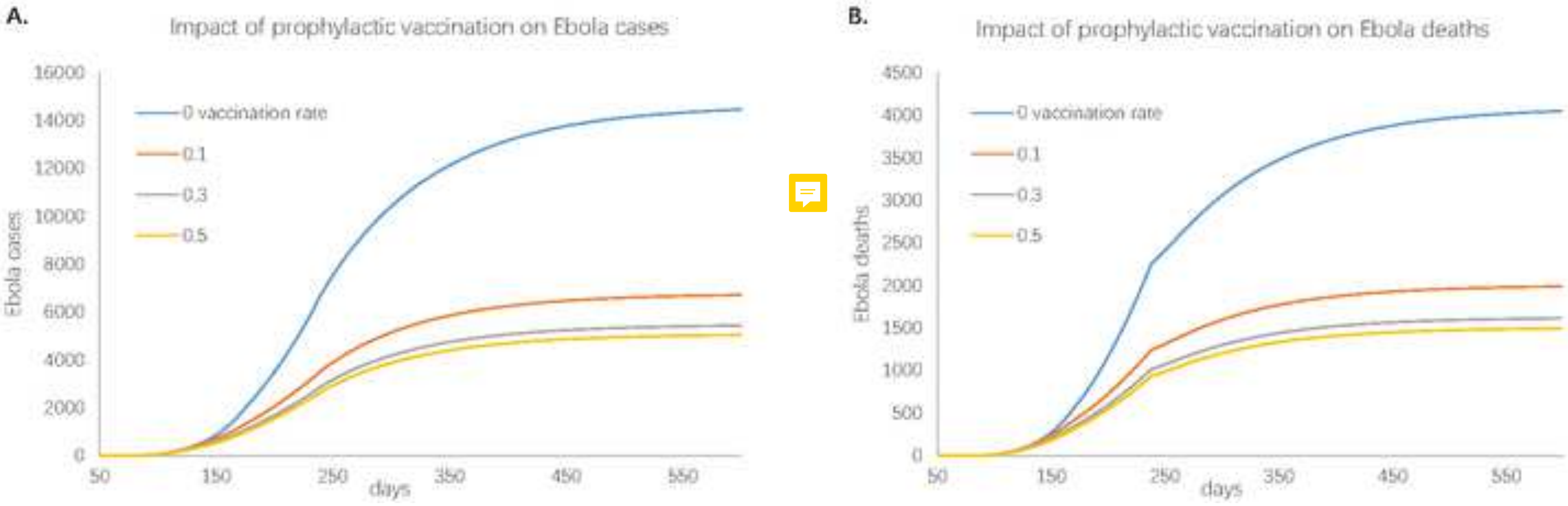

Click here to access/download;Figure;fig10.tif

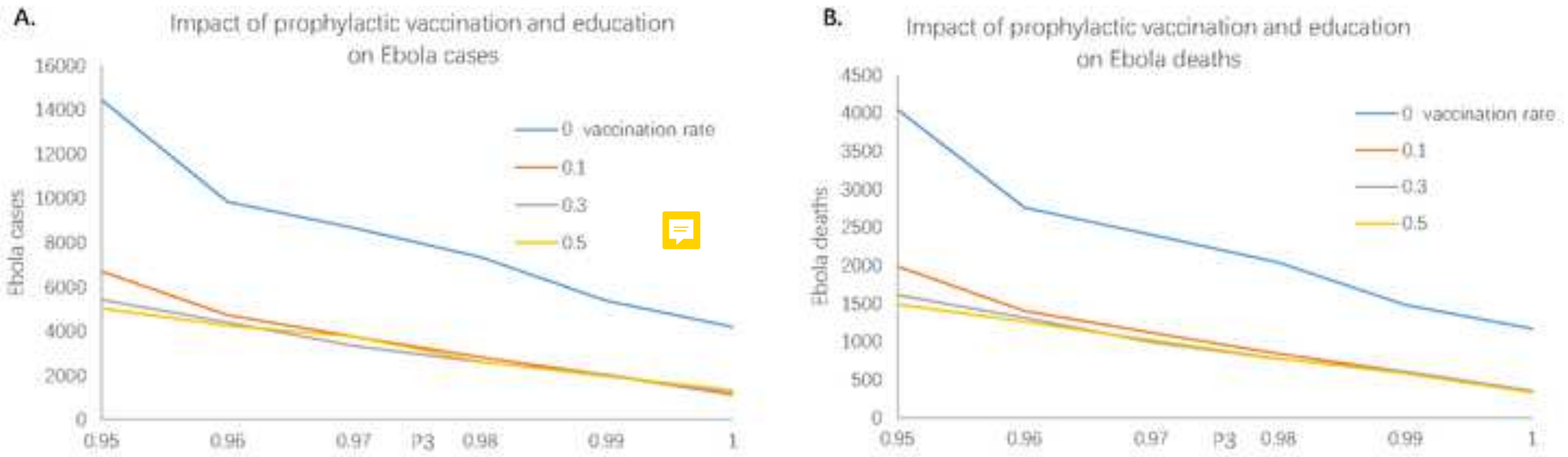

Click here to access/download;Figure;fig11.tif

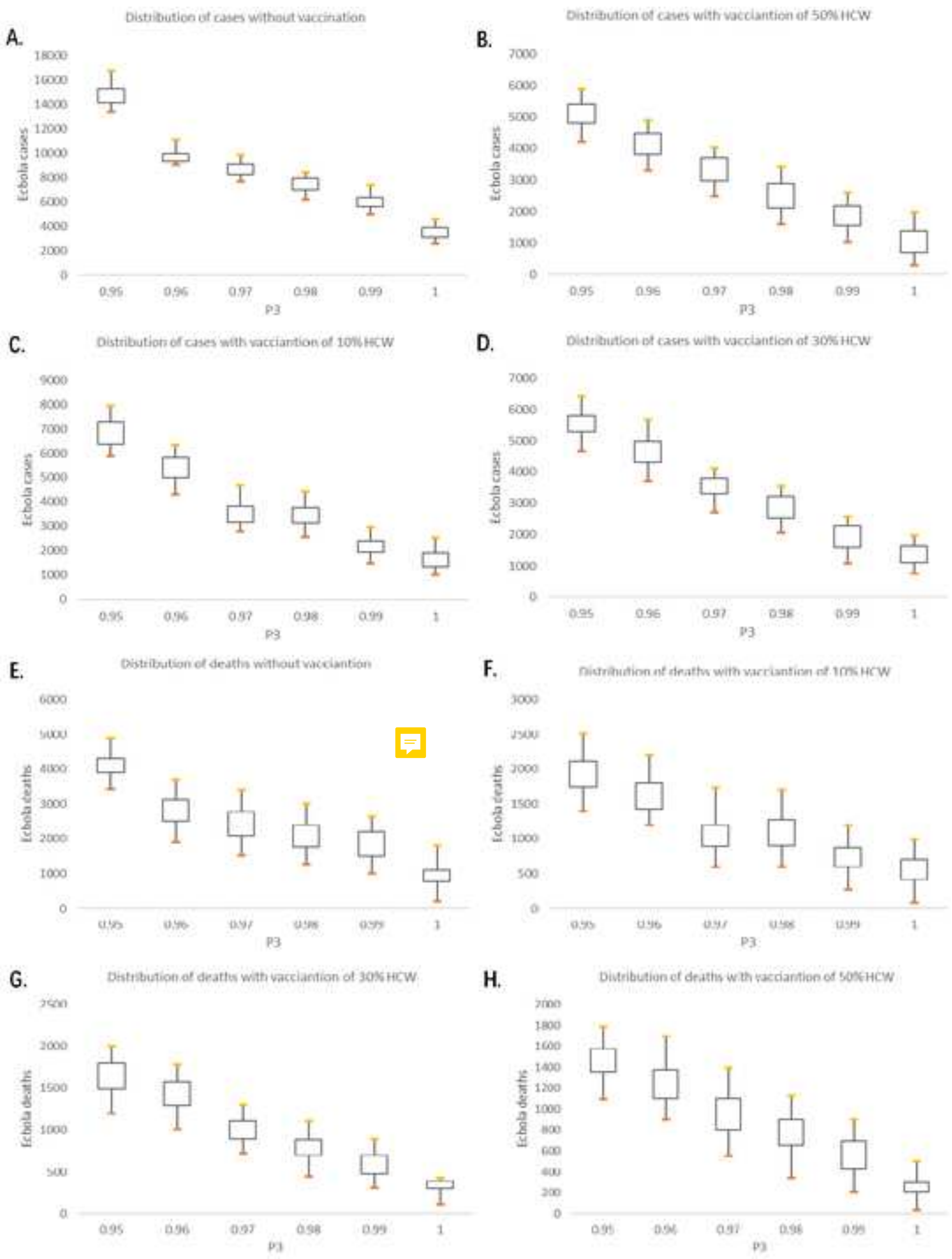

Click here to access/download;Figure;fig12.tif

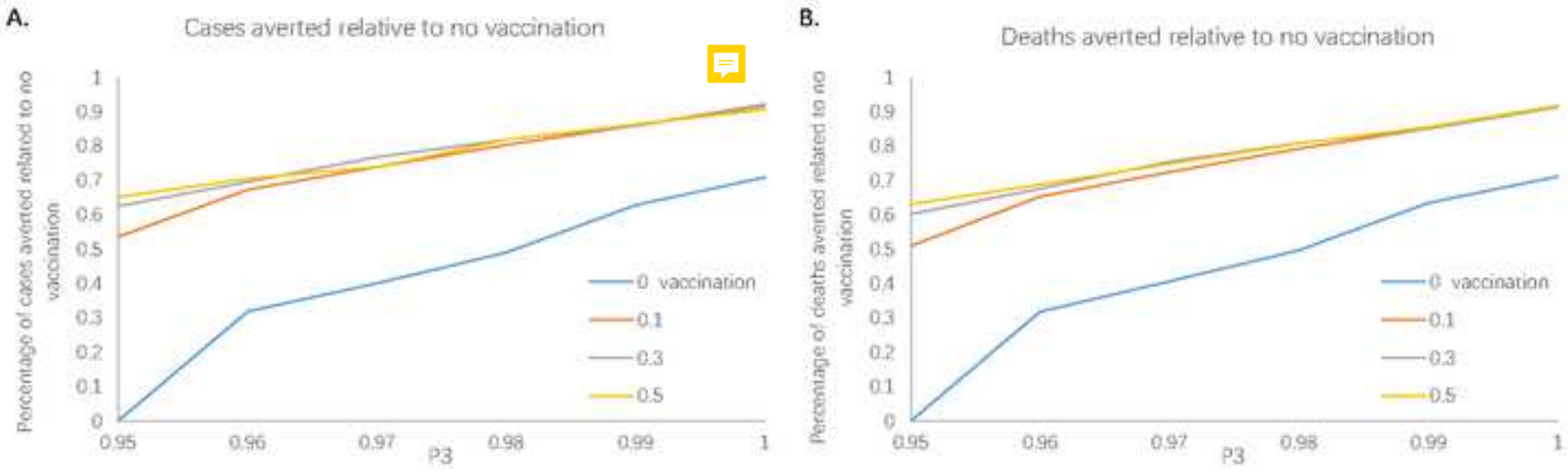

Click here to access/download;Figure;fig1.tif

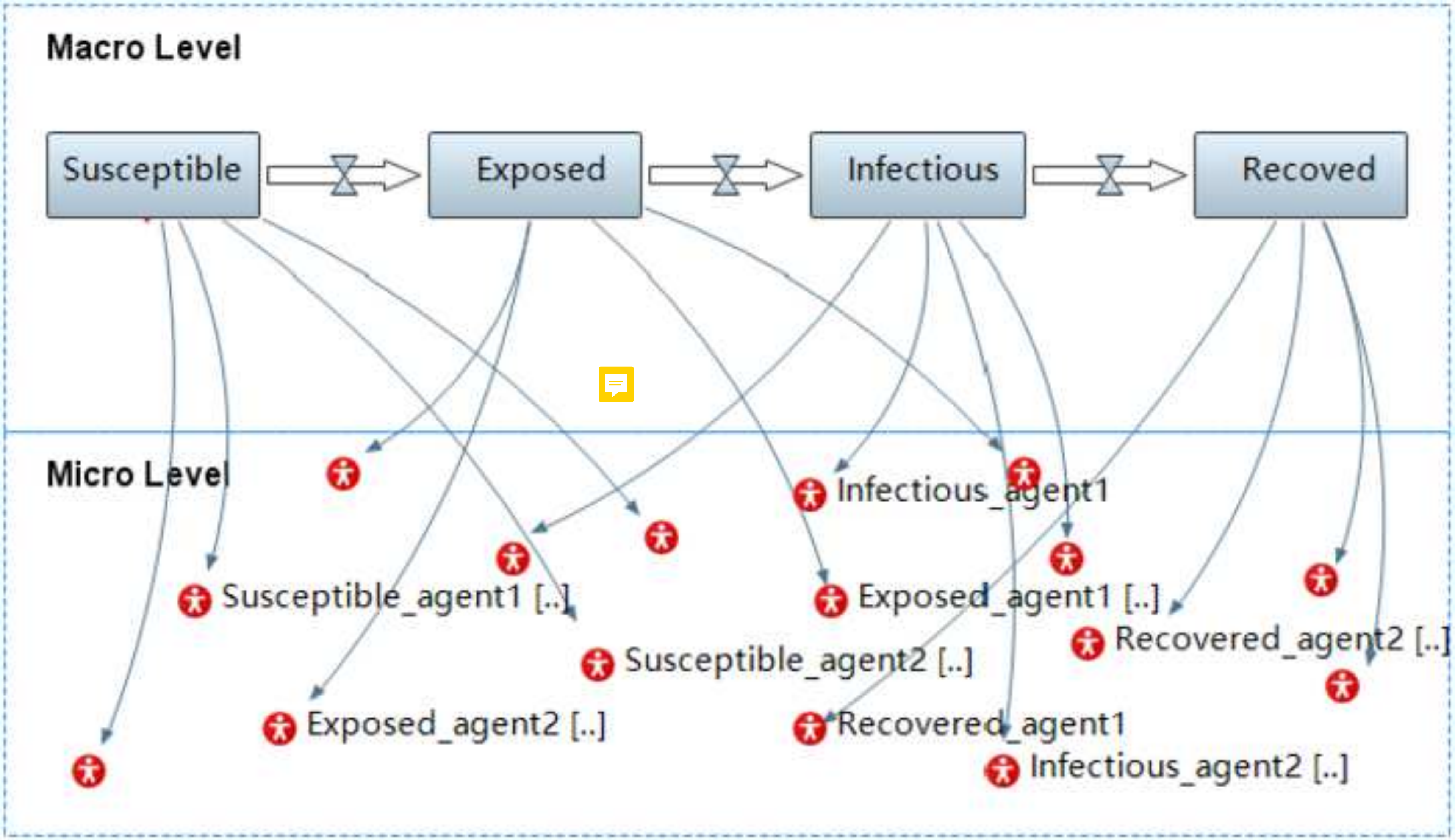

Click here to access/download;Figure;fig2.tif

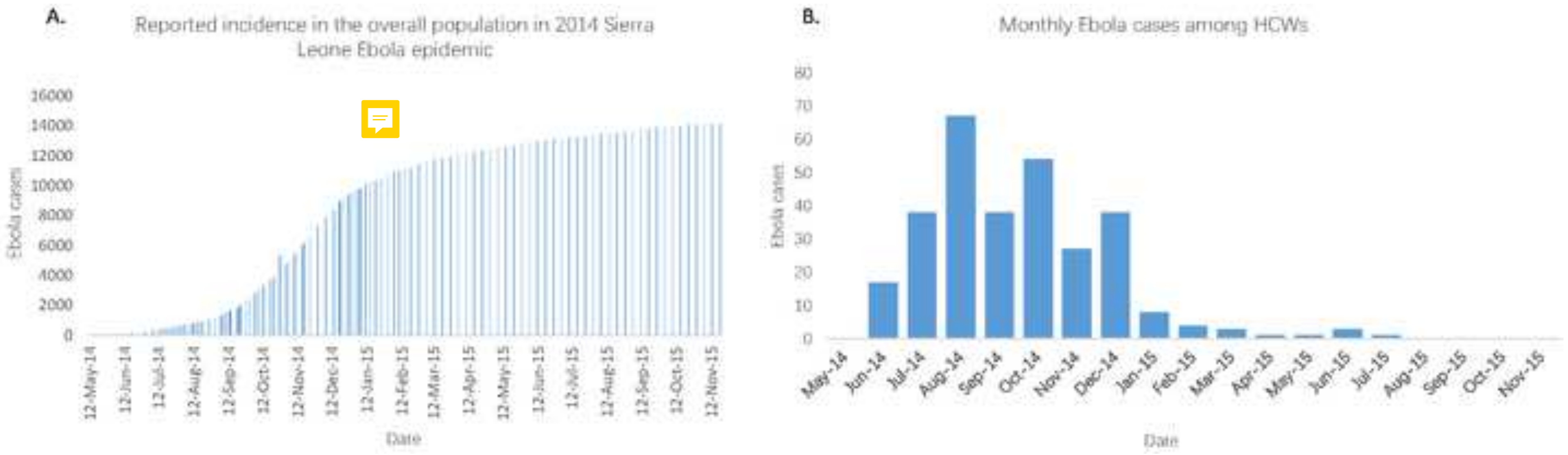

Click here to access/download;Figure;fig3.tif

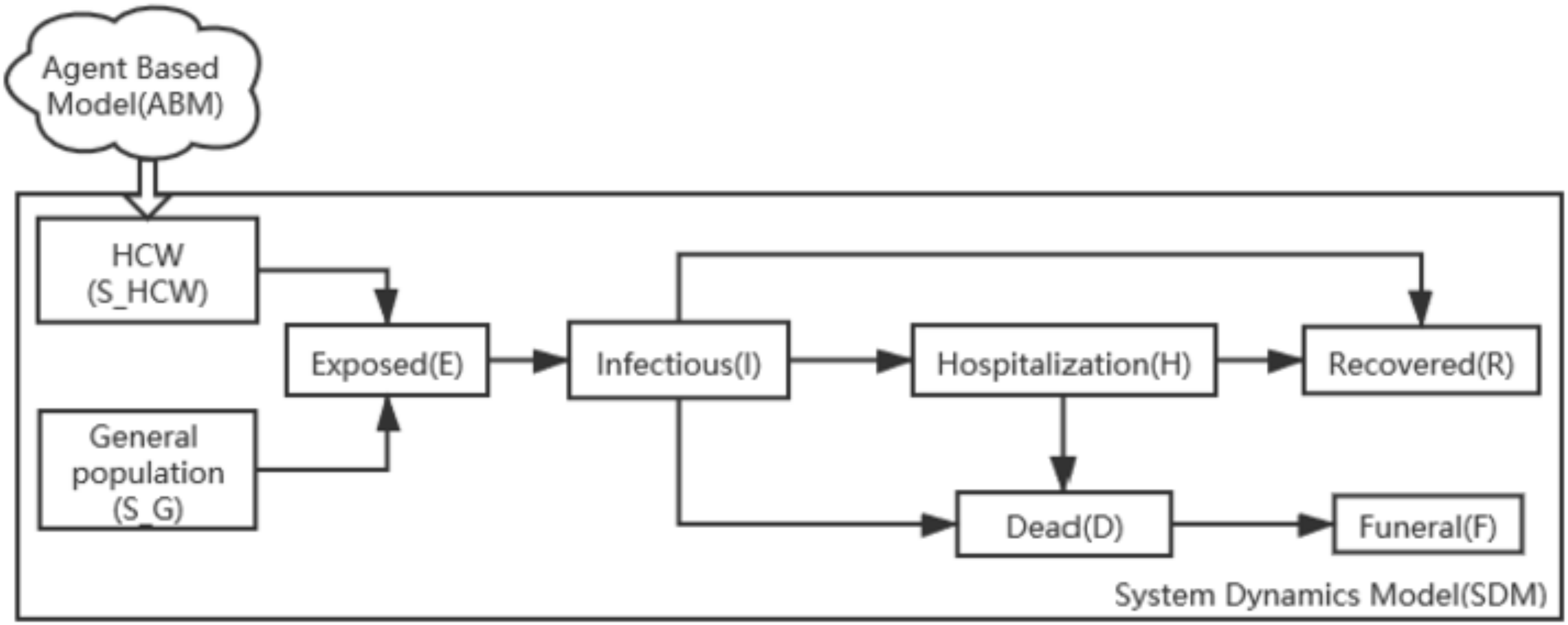

Click here to access/download;Figure;fig4.tif

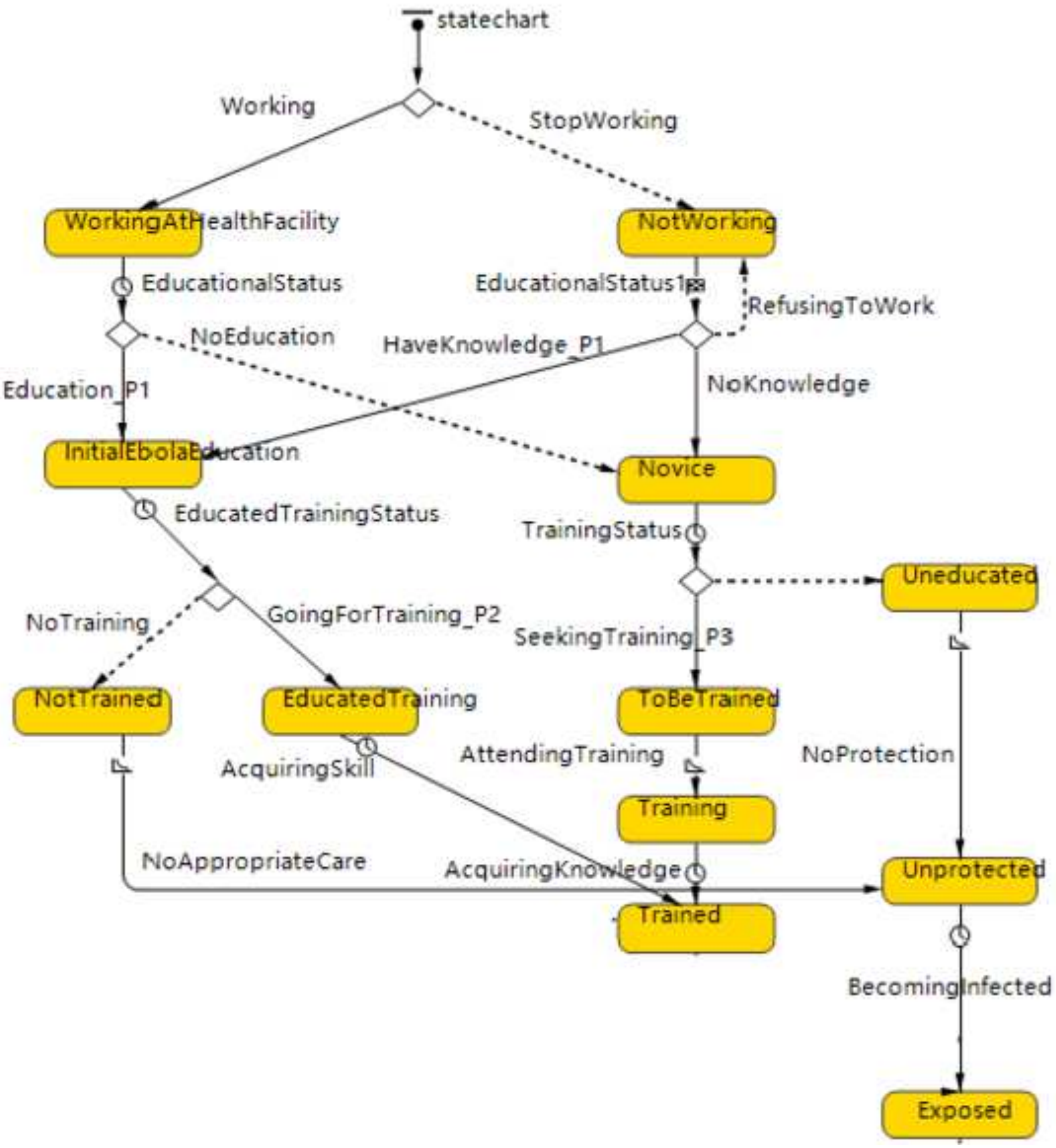

Click here to access/download;Figure;fig5.tif

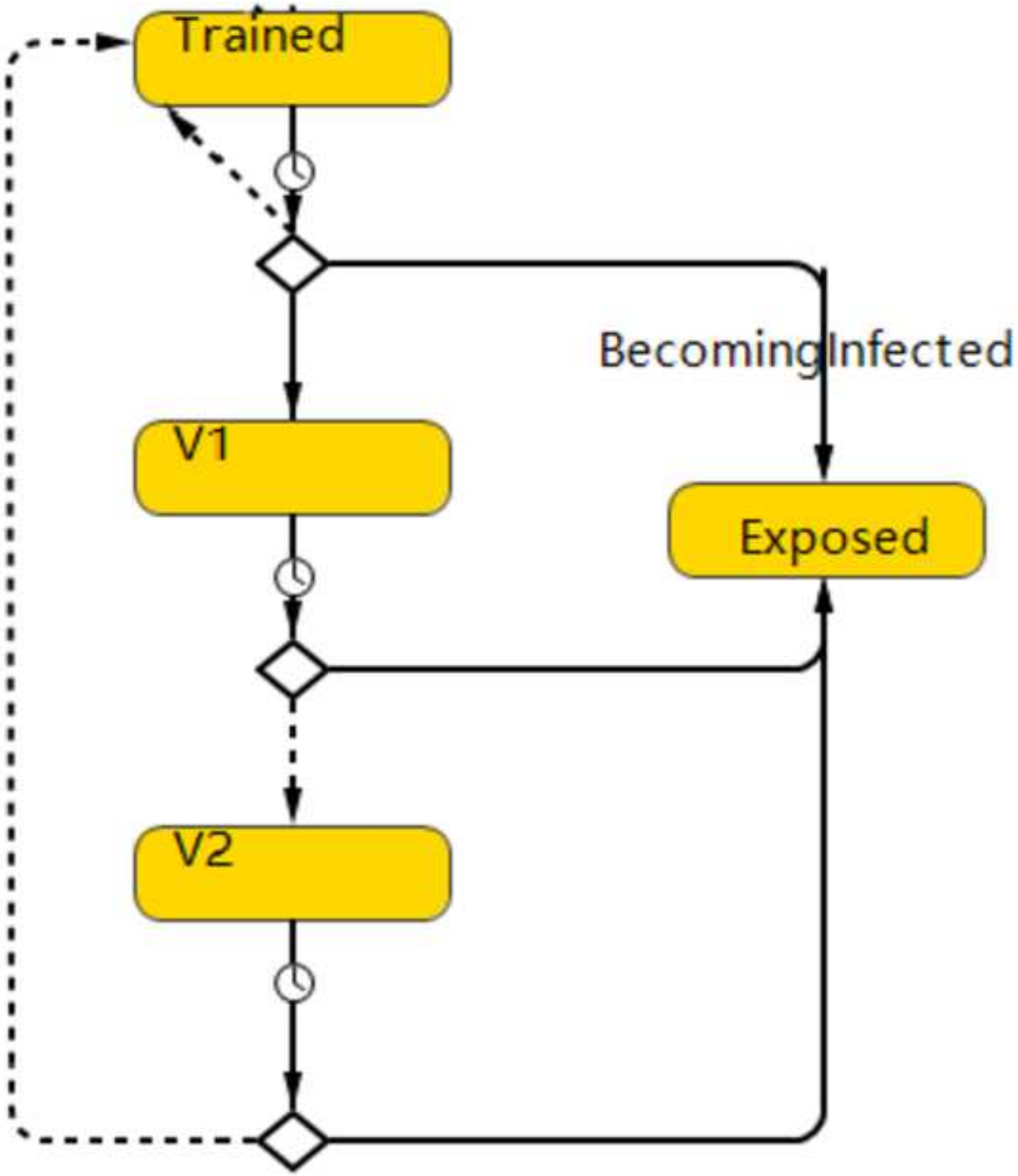

Click here to access/download;Figure;fig6.tif

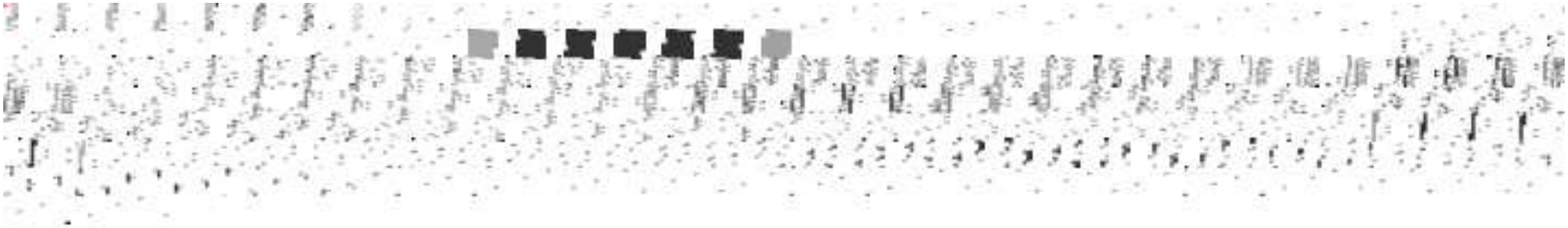

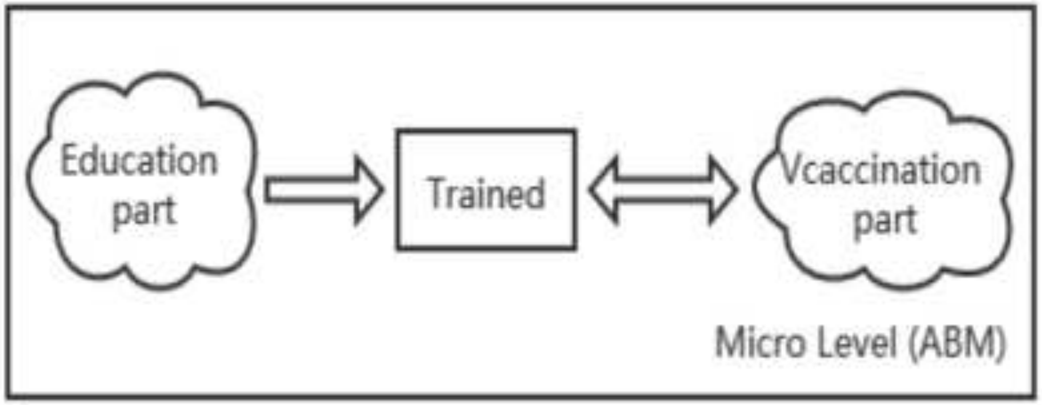

Click here to access/download;Figure;fig7.tif

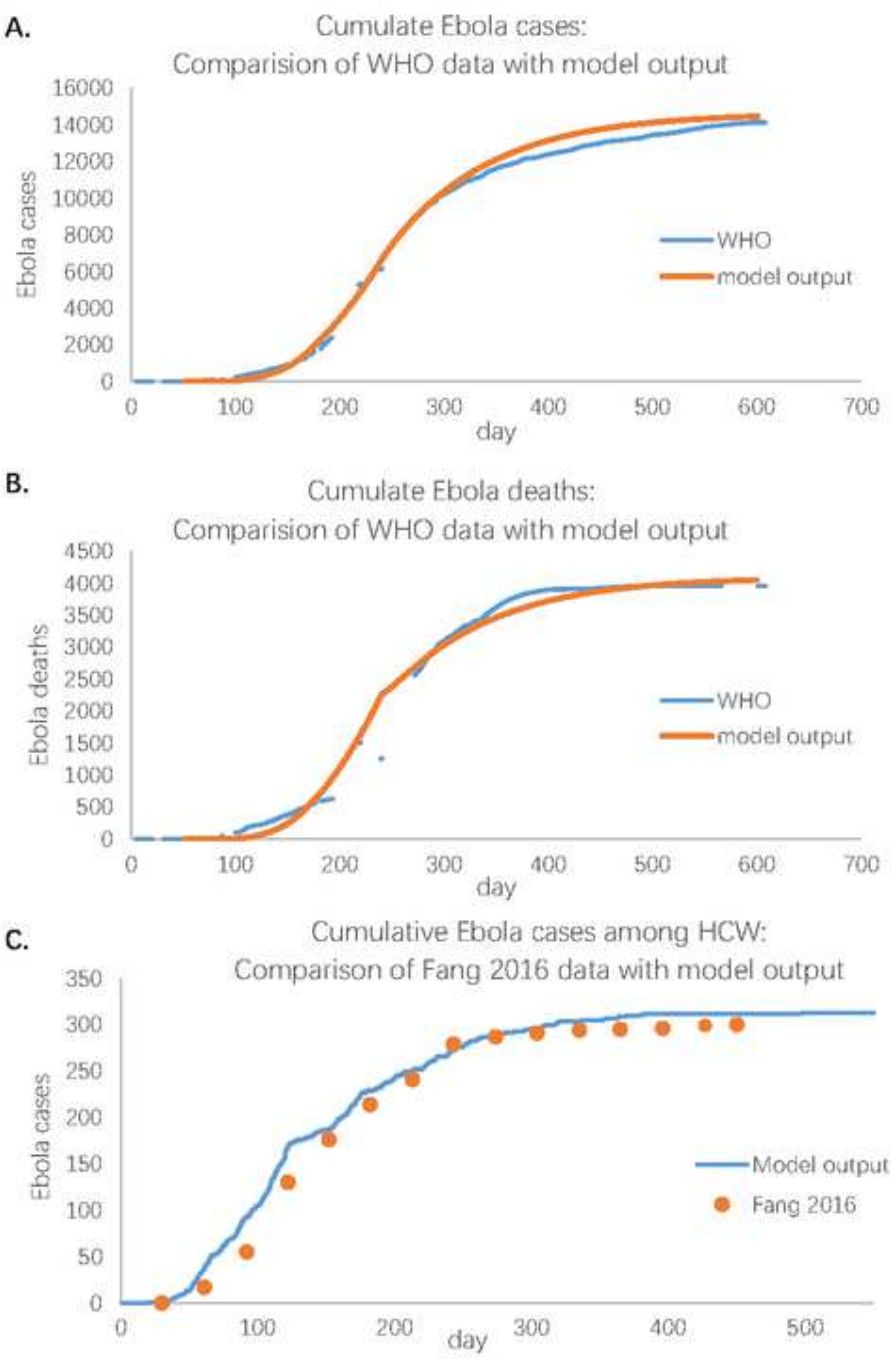

Click here to access/download
Supporting Information
S1_file.docx
