# Supplementary material for: A hybrid simulation model to study the impact of combined interventions on Ebola epidemic
Source: PLoS One. 2021 Jul 6;16(7):e0254044. doi: 10.1371/journal.pone.0254044 (PMC8259970; doi:10.1371/journal.pone.0254044)
Supplement: Attachment — Submitted filename: Response to reviewers.pdf [file pone.0254044.s003.pdf]

# Reply to the Editors Comments and Reviewers on Paper PONE-D-20-38313

Submission to PLOS ONE

April 21, 2021

## 1 Response to Editors

Thanks for reviewing our manuscript and providing your insightful suggestions concerning "A hybrid simulation model to study the impact of combined interventions on Ebola epidemic" (PONE-D-20-38313). Those comments are all valuable and very helpful for revising and improving our manuscript, as well as the important guiding significance to our researches. We have studied comments carefully and have made correction which we hope meet with approval. The revised portion is marked in blue in the paper. The following is our response for your additional requirements.

*Comment:* Please ensure that your manuscript meets PLOS ONE's style requirements, including those for file naming.
*Reply:* Thank you for reminding. Our manuscript has already typeset with PLOS ONE style templates.

*Comment:* Please ensure you have thoroughly discussed any potential limitations of this study within the Discussion section, including the potential impact of confounding factors.
*Reply:* Thank you for the kind reminding. We have discussed the limitations of our work in "Discussion".

*Comment:* Please remove any funding-related text from the manuscript and let us know how you would like to update your Funding Statement.
*Reply:* Thanks for the reminder. We have removed the funding-related text from the manuscript. We feel sorry for submitting the wrong financial disclosure in the initial submission. According to the "Submission Guidelines", we ensure that we received no specific funding for this work and we apply for changing the financial disclosure.

*Comment:* Please revise the manuscript to rephrase the duplicated text, cite your sources, and provide details as to how the current manuscript advances on previous work. Please note that further consideration is dependent on the submission of a manuscript that addresses these concerns about the overlap in text with published work.
*Reply:* Thanks for the reminder. We revised our manuscript and rephrased those text overlaps with published works.

In the following, the points raised by the reviewers will be addressed in detail, respectively. We would like to express our sincere thanks to you and reviewers for the constructive and positive comments and we look forward to your information about our revised manuscript.

Yours Sincerely

Peiyu Chen

## 2 Response to Reviewer #1

Comment: The authors use the term "system dynamics model" for SEIR models. The usual term used for such models is compartmental or mechanistic dynamical models. I don't insist that they change the terminology, but this is more appropriate.
Reply: Thanks for your helpful advice. It is indeed necessary to clarify the definition of system dynamic model and SEIR model. We carefully confirmed the definition of SEIR model which is indeed not a system dynamic model. In the revised manuscript, we looked through the related parts and the statement of "system dynamic model (SDM)" were corrected as "mean-field compartmental model" or "SEIR dynamic model" through the whole manuscript.

Comment: The authors should elaborate more in the use of English. There are several syntactical errors. Typos in Line 16.
Reply: Thanks for your kind suggestion. We revised long and incomprehensible sentences in the whole manuscript and tried our best to scan the whole manuscript to eliminate typos and grammatical errors. We hope the English writing can be improved.

Comment: Line 18: "Furthermore, Tsanou et al. [9] studied the potential impact of 18environmental prophylactic vaccine" The word vaccine is misleading and should NOT be used in that way. Tsanou et al. studied the effect of environmental contamination and how adequate hygienic living conditions could affect the spread.
Reply: Special thanks to you for your good comments. We verified the study [9] (in previous manuscript) and replaced this reference with reference[8] (in the revised manuscript) for reviewer 2 pointed out that it is a wrong referencing. Line 16-18, we state that " Furthermore, Berge T et al. [8] studied the effect of vaccination and self-protection measures in the transmission dynamics of Ebola in Africa." Thanks anyway for pointing out our mistake.

Comment: Line 20: following up the previous statement they state that "However, the stochastic nature of the movement of the individual is not considered explicitly with SDM for they only describe the mobility dynamics of sub-populations [10]." This sentence is vague and needs more elaboration. What do they mean with "for they only describe the mobility dynamics of sub-populations?" The model in Tsanou it is a compartmental model and not an individual-based model? This is what they mean?
Reply: Thank you for pointing out this error. We are very sorry for misquoted their studies. In the updated manuscript, we replaced the reference and rewrote this part. Line 16-20, we discussed reference [8] in the answer above and pointed the shortcoming of the mean-field compartmental model.

Comment: Line 27. The authors state "However, it is tough to gain an insight of the epidemic dynamics 27with vole populations through ABM due to the massive calculations.". The main difficulty when using ABM is not the massive calculations, but (1) the uncertainty in calibrating the many parameters and variables, the connection to the compartmental sub-group scale in a systematic way, the uncertainty in modeling the underlying high dimensional contact network. Computational cost is secondary.
Reply: Thanks for your suggestion. As you stated, it is really true that the uncertainty of the ABM output is more essential than it computational limitation. So, we rewrote the reason for ABM's difficulty of applying in large scale population. Line 26-29, we state that "The randomness of ABM's output leads to the uncertainty in calibrating parameters and the sophisticated contact network. Therefore, when modeling large scale population, it is tough to gain an insight into the epidemic dynamics through ABM."

Comment: A general comment about the review of the literature: There is a huge literature in epidemi-

ology about agent-based models that have been used for various purposes. A relatively small subgroup of these have been developed for describing the Ebola epidemic in West Africa. They authors cite only a very small number of papers related to agent-based modelling but also not related to the epidemic of Ebola. Thus they have to elaborate more on the review of the existing literature, and they have to cite and discuss key papers that have introduced agent-based models that have focused on the Ebola dynamics.
Reply: Thanks for your advice. We checked the recommended references, which are indeed relevant and helpful. In the revised version, we cite all the suggested papers in the section of introduction and the section of reference is also updated, mainly covering [12], [23], [24] and [29]. We discussed the study[12] in the introduction for ABM (Line 24-26) and the other tree studies in the introduction for EVD and its interventions using ABM (Line 48-52, Line 62).

Comment: The description of the modeling approach needs a lot of improvement. Actually the agent-based model is only described by a schematic and a table at the supporting information containing the transitions in the micro level.
Reply: Thanks for your insightful suggestion. According to your suggestion, we reorganized the "Model" part, redrew the schematic diagram for the hybrid model(Fig 1 in response) and added description for the ABM and its linkage with the SEIR model. In the previous version of the manuscript, we described the modeling method (Hybrid modeling and simulation method, fig1) and the model (SEIR model and agent-based model, fig3, 4, 5, 6) separately.

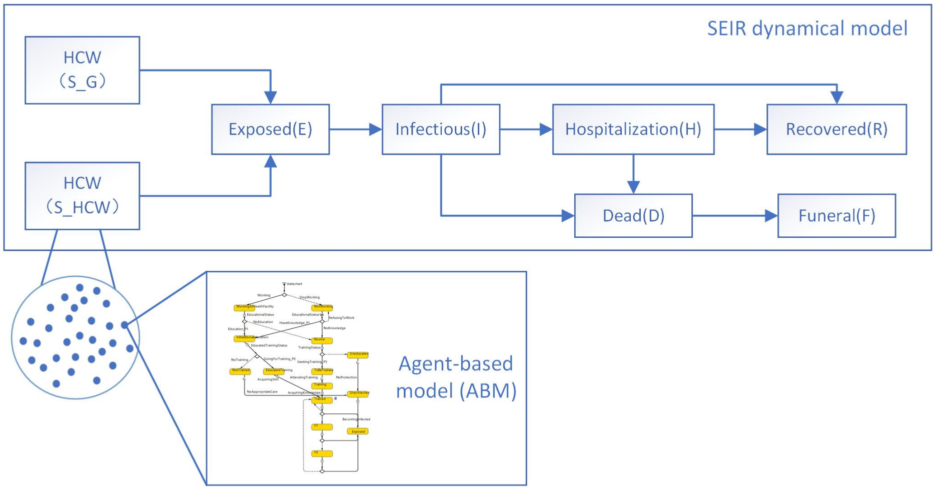


Fig. 1. Hybrid simulation model

In the updated manuscript, to explain the model explicitly, we described the method and the model together in the subsection "Hybrid modeling and simulation method and the model". Besides, we replaced fig1 and fig3 (the previous version) with Fig 1 (fig2 in the new version), a more specific schematic diagram, to illustrate our model structure. Fig 1 shows the combination of SEIR model and agent-based model (ABM) and their modeling objects respectively. In our model, ABM is built only for susceptible heath care works (HCWs) and do not consider the disease transmission dynamic. For the susceptible HCWs are not capable of infecting others, we focus on the details of HCW's social activities relating to the interventions and their roles in disease dynamics are depicted in the macroscope model. In summary, we have the following major revisions about the description of ABM in the new version.

- ABM and SEIR model run in parallel. Our method handles stochastic events such as vaccination and pre-deployment training and continuous processes like the spread of virus at the same time. The global model runs in hybrid time with epidemic dynamics calculated in continuous time, and individual state

changes and their related processes (training, vaccination stage, decision to work) occurring as discrete asynchronous events.

- Functions of SEIR model and ABM. The hybrid model can reproduce the viability of a susceptible HCW in a heterogeneous environment by coupling a decision making submodel in ABM, with a disease transmission in SEIR model. ABM is applied to gain an insight into the the group of susceptible HCW in the SEIR dynamic model. Each agent has an associated social state related to their reaction towards education or vaccination interventions. The agent makes decisions depending on its current condition and related transition rules. The contact infection among individuals is considered by the SEIR mean-field compartmental model, therefore, we do not build contact network in ABM.

- Description for the linkage between these two parts. The output of the ABM is the number of exposed HCWs calculated by the parameters and variables gathered in the SEIR model (see in S1 File). And the exposed HCWs, in return, join in the dynamics of virus transmission in the SEIR model.

The detailed descriptions and definitions can be referred in the subsection "Hybrid modeling and simulation method and the model" (Line 131-154) and the section "Agent-based model" (Line 175-194). The schematic diagram can be referred in fig2 in the revised manuscript.

*Comment:* Many of the parameters in the table of the SI appear as ad-hoc (70% possibility etc). These values can be justified.
*Reply:* Thanks for your advice. It is really true as reviewer suggested that we did not fully explain and verify the value of parameters. We consulted relevant literature and verified some parameters mentioned in the table of the S1 File. The data in red (S1 file verified) were verified according to the study or discussed by additional experiments. We gave detailed description as followed.

- Working at health facility: 70%; Initial Ebola education: 10%

  The rate for working at health facility and initial Ebola education are referred to [1]. The respondents in this research are HCWs in Ashanti Region of Ghana. We used their research results since the capacity of health systems in Ashanti Region of Ghana and Sierra Leone are similar. Besides, it is the closest data we could obtain.

- Pre-deployment training time: 3 days for HCWs with initial education and 5 days for HCWs without initial education; Training speed: 5 person per day

  We obtained the education-related parameters according to the study reported by Jones-Konneh TEC [2]. However, there is a discrepancy between the number of HCW for the difference of composition of HCW ("frontline works" mentioned in [2]). So we did equal scaling for the relevant parameters.

Table 1. Sensitive analysis for parameters

| parameter | 0% | 10% | 20% | 30% | 40% | 50% | 60% | 70% | 80% | 90% | 100% |
|---|---|---|---|---|---|---|---|---|---|---|---|
| P1 | 5.8% | 6.3% | 6.9% | 7.4% | 8.1% | 9.0% | 9.9% | 10.8% | 11.1% | 11.9% | 12.7% |
| P2 | 18.0% | 15.9% | 15.0% | 14.5% | 13.9% | 11.9% | 11.6% | 9.6% | 8.0% | 7.0% | 6.2% |
| P3 | 88.3% | 80.4% | 72.1% | 63.0% | 55.4% | 45.6% | 37.9% | 28.0% | 20.3% | 10.9% | 2.1% |

- There are some parameters that could not be supported by the existing research. So, we estimated these parameters according to the studies [3, 4, 5] and proved that the estimation error of these parameters has little effect on the experimental results. We did sensitive analysis for these parameters including the pre-deployment training rate which has been further studied in our paper. Here, P1 means "initial Ebola

education", P2 means proportion of "educated training" and P3 means proportion of pre-deployment training (S1 File) . To evaluate the sensitivity of these parameters, we focused on the output of ABM. We counted the number of agents which became "Exposed" and calculated the proportion of HCWs being exposed. The result is shown in Table 1. Based on Table 1, we concluded that the influence of P1 and P2 on model output is relatively small while P3 has great influence on model output. Therefore, the deviations of the value of P1 and P2 have little effect on the model output.

*Comment:* The authors should describe this in detail. A prominent characteristic of agent-based models is the underlying social-transmission network. There is no such information in the presentation of the model. Without such a heterogeneity, i.e. when the interactions are uniform and random, an agent –based mode can be easily be substituted by a mean field model.
*Reply:* Thanks for your insightful comments. As you stated, it is true that we do not build social-transmission network in the ABM for we only focus on the uninfected HCWs who do not contribute to virus transmission and the contact infection has already considered in the SEIR model. Although the ABM's feature of building social-transmission network is not realized in our work, we used other characteristics of ABM.

ABM's benefits can be generalized in three statements: i. ABM captures emergent phenomena; ii. ABM provides a natural description of a system; and iii. ABM is flexible.[6] In our study, multiple interventions such as education and vaccination need to be considered. The HCW's behavior is nonlinear and can be characterized by chance or if-then rules. It is difficult to describing discontinuity in individual behavior with aggregate flow equations. Therefore, ABM is used for its ability to deal with such system. Besides, ABM is natural for describing and simulating our system for each parameter has intuitive meaning and the adjustment of the value has guiding significance to government policy making. The detailed explanation can been seen in Line 173-184.

*Comment:* The same hold true for the calibration. There is no information about the algorithm that has been used to fit the parameters of the model and because of the fact that the agent-based model is not well described
*Reply:* Parameters in the ABM are fixed and their values are determined as close to reality as possible according to the prior knowledge [7, 8, 9, 10]. Therefore, the free parameters ("Fitted" parameters listed in Table1(in the revised manuscript)) that require calibration are in the SEIR dynamic model. We develop the hybrid model using the multi-method simulation software AnyLogic Professional (version 8.3.1) [11] and do calibration by "Calibration Experiment" (Monte Carlo experiments) in AnyLogic. The calibration experiment calculates the difference between the simulation output and the given data and returns a non-negative value which is a square root of the average of square of difference between sets of data. By running 3000 iterations of the calibration experiment, we obtain the minimal value and the corresponding parameters, as it means the least difference between two sets of data. In addition, we set fixed intervals for the free parameters during calibration referring to the parameters reported by Potluri R [12].

The detailed descriptions can be referred in the subsection "Model parameterization and validation"(Line 255-269) Besides, we added the 95% CI for all the model output in S1 File and the "Result"(Line 277-278).

## 3 Response to Reviewer #2

*Comment:* Wrong referencing: Please correct it by some or all the following references.
*Reply:* We sincerely appreciate the valuable comments. We have checked the recommended literature carefully, which are indeed relevant and helpful. We changed the wrong reference and added more references on in the section of introduction. In the updated paper, we have cited three of the suggested papers. We replaced the

original reference [9] (in the previous manuscript) with reference [8] (in the revised manuscript) and added reference [19] in line 33-35 and reference [4] in line 8 and gave brief discussions for these studies.

Comment: This ABM method is not specific for this work. Why didn't you use Fig.3 to illustrate the method? That will be more relevant for the current work. Comments on fig 1: this micro level model does not take into consideration the two different types of susceptible individuals (HCW and general community)

Reply: Thanks for your insightful suggestion. According to your comments, we reorganized the "Model" part, redrew the schematic diagram for the hybrid model and added up description for the ABM and its linkage with the SEIR model. In the previous version of the manuscript, we described the modeling method (Hybrid modeling and simulation method, fig1) and the model (SEIR model and agent-based model, fig3, 4, 5) separately. In the updated manuscript, to explain the model clearly, we described the method and the model together in the subsection "Hybrid modeling and simulation method and the model". Besides, we replaced fig1 and fig3 with fig2(Fig 1 in this response), a more specific schematic diagram, to illustrate our model structure. Fig 1 shows SEIR model and agent-based model (ABM) and their modeling objects respectively. In our model, ABM is built only for susceptible heath care works (HCWs) and do not consider the difference with the general population. For the susceptible HCWs are not capable of infecting others, we focus on the details of HCW's social activities relating to the interventions and their roles in disease dynamics are depicted in the macroscope model. In summary, we have the following major revisions about the description of ABM in the new version.

- ABM and SEIR model run in parallel. Our method handles stochastic events such as vaccination and pre-deployment training and continuous processes like the spread of virus at the same time. The global model runs in hybrid time with epidemic dynamics calculated in continuous time, and individual state changes and their related processes (training, vaccination stage, decision to work) occurring as discrete asynchronous events.
- Functions of SEIR model and ABM. The hybrid model can reproduce the viability of a susceptible HCW in a heterogeneous environment by coupling a decision making submodel in ABM, with a disease transmission in SEIR model. ABM is applied to gain an insight into the the group of susceptible HCW in the SEIR dynamic model. Each agent has an associated social state related to their reaction towards education or vaccination interventions. The agent makes decisions depending on its current condition and related transition rules. The contact infection among individuals is considered by the SEIR mean-field compartmental model, therefore, we do not build contact network in ABM.
- Description for the linkage between these two parts. The output of the ABM is the number of exposed HCWs calculated by the parameters and variables gathered in the SEIR model (see in S1 File). And the exposed HCWs, in return, join in the dynamics of virus transmission in the SEIR model.

The detailed descriptions and definitions can be referred in the subsection "Hybrid modeling and simulation method and the model" (Line 131-154) and the section "Agent-based model" (Line 175-194). The schematic diagram can be referred in fig2 in the revised manuscript.

Comment: Why splitting the simulation period into three different intervals ? What motivates this choice ? Which population characteristics motivated this splitting ?

Reply: Thanks for your question. It would be more understandable if we add explanation for these questions. The related statement in the revised draft is as follow (referred to Line 109-114): We split the simulation period based on the characteristic of the increasing trend of the Ebola cases. According to the reported data shown in Fig 1, the entirety of the simulation horizon (March 22,2014 to November 13,2015) was divided into three periods: a) In the first 123 days, a sharpen increase in the reported Ebola cases among HCWs per month

and a relatively slower increase in cases among the overall population per week were shown. b) In the second period (123days to 190 days) a decline in cases among HCW and an increase in cases among the general population were witnessed. c) In the third period (190 days to 550 days), there can be seen a decline both in cases among HCW and the general population. This three-stage decomposition can also be explained that the evolution of the epidemic will always experience early phase, middle phase, late or recovery phase, each phase has their own epidemic size transition characteristics. Therefore, the three-stage decomposition also correspond to the three phases in the Ebola epidemic. This decomposition method can also be seen in other works [12, 13].

Comment: Unrealistic assumption for an still experimental vaccine. Why was the education training broadened to the general community ? what prevents this to be done ?
*Reply:* Thanks for your questions. This two questions are both raised for the section "Model assumption", therefore, we give our explanation together in this paragraph. As for vaccination rate, Henao-Restrepo et al. [14, 15] demonstrated 100% (95% confidence interval (CI): 74.7–100.0) efficacy of the rVSV-ZEBOV vaccine against EVD with a total population of 7651 people included in the planned analysis in July 2015. Although the vaccine has not been popular yet, the efficiency of the vaccine was verified in a certain size of population. It is the most authoritative data, indicating the efficacy of the rVSV-ZEBOV vaccine against EVD, available currently. Taking the above into consideration, we keep the assumption for vaccination efficacy unchanged. Thanks anyway for giving the suggestion.

In our paper, "education" refers to the pre-deployment training focusing on protecting first responders through disease-specific information and safety measures and preparing them for a range of specific Ebola response functions, including treatment of Ebola patients, case tracking, safe burials, epidemiology and infection prevention and control. A review [16] reported by WHO was conducted to understand the impact of training for first responders in the Ebola emergency response and explained the reason why it is essential to complete specific trainings prior to deployment. Here, we did not consider the education for the overall population since carry out such large-scale education is time consuming and costly. Besides, the lack of supporting data with respect to the universal education makes it difficult to take this situation into consideration. However, it is well worth considering in our future study.

Comment: Please indicate how the calculation of the averted cases were done.
*Reply:* Thanks for your question. Proportion of cases averted vs no intervention =

$$\frac{CCWOI - CCWI}{CCWOI} * 100\% \tag{1}$$

CCWOI means cumulative cases without intervention and CCWI means cumulative cases with intervention. In other words, the "proportion of cases averted vs no intervention" indicates the ability to interrupt the spread of the epidemic.

Comment: Is there any reason why this increment (20%) should be so high as compared to the increment of pre-deployment training (1%) ? Is the answer is No, then I believe that to get smoother figures (Figs 10- 12) and avoid inflexion points, you should use an increment of 10%. In fact, the possible correlation between Pre-deployment training and Vaccination rate should guide these increments. If you opt for any change in the increments, please remember it will have consequences on the related Tables (5 and 6) and results which you must update accordingly.
*Reply:* Thanks for your insightful suggestion. Considering the your suggestion, we did additional experiment for our work. According to Table 5, we can see that under the same pre-deployment training rate, the gaps of aversion rate between 10%, 30% and 50% vaccination proportion are below 2%. However, under the same vaccination rate, the gaps of aversion rate between different pre-deployment training are about 8% which are more remarkable than the former ones. If we set the increment to be 10%, the gaps of aversion rate would be

smaller than 1%. Besides, the polylines in Fig 11(in the revised version) representing the intervention of 10%, 30% and 50% are already closed, if we further take the 20% and 40% vaccination rate into consideration, the polylines can be indistinguishable.

In terms of smoothing of the curve (Fig 9 and Fig 11 in the revised version), it is more effective to use 0.5% increment for pre-deployment training rate than 10% increment for vaccination rate. However, the 1% increment in pre-deployment training rate correspond to 11.53 HCWs, we thought the precision is high enough for the government policy decision-making. Hence, in the updated manuscript, we use 0.5% increment in pre-deployment training rate to optimize Fig 2 (Fig 9 in revised manuscript) and Fig 3 (Fig 11 in revised manuscript) but do not consider the additional cases in our research.

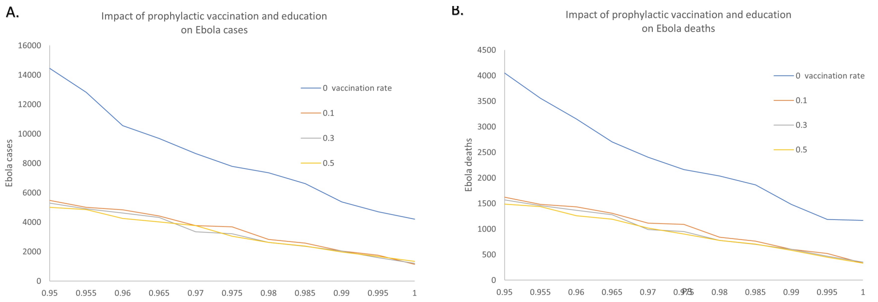


Fig. 2. Impact of combined interventions on cumulative Ebola cases and deaths

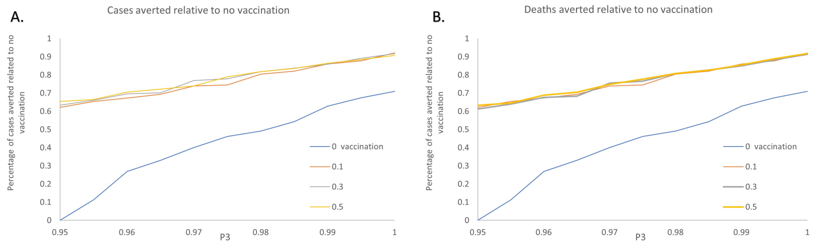


Fig. 3. Impact of combined interventions on aversion rate compared with no vaccination scenario

<u>*Comment:*</u> Make short and precise sentences; The level of English is not good at all in paragraph, and makes things very difficult to capture: Please, make short and precise sentences.
<u>*Reply:*</u> Thanks for your kind suggestion. We rewrote the paragraphs you had marked, revised long and incomprehensible sentences in the whole manuscript and tried our best to scan the whole manuscript to eliminate typos and grammatical errors. We hope the English writing can be improved.

<u>*Comment:*</u> Bad resolution.
<u>*Reply:*</u> Thanks for pointing out our mistake. We improved the picture's clarify for all the figures(fig1-fig11) to meet PLOS requirements.

# References

[1] A. A. Annan, D. D. Yar, M. Owusu, E. A. Biney, P. K. Forson, P. B. Okyere, A. A. Gyimah, and E. Owusu-Dabo, "Health care workers indicate ill preparedness for ebola virus disease outbreak in ashanti region of ghana," *BMC Public Health*, vol. 17, no. 1, p. 546, 2017.

[2] T. E. C. Jones-Konneh, A. Murakami, H. Sasaki, and S. Egawa, “Intensive education of health care workers improves the outcome of ebola virus disease: Lessons learned from the 2014 outbreak in sierra leone,” *The Tohoku Journal of Experimental Medicine*, vol. 243, no. 2, pp. 101–105, 2017.

[3] H. Wurie and S. Witter, “Serving through and after conflict: life histories of health workers in sierra leone,” *REBuild Consortium*, 2014.

[4] J. Raven, H. Wurie, and S. Witter, “Health workers' experiences of coping with the ebola epidemic in sierra leone's health system: a qualitative study,” *BMC health services research*, vol. 18, no. 1, pp. 1–9, 2018.

[5] UNICEF, “Sierra leone health facility survey 2014 - assessing the impact of the evd outbreak on health systems in sierra leone - survey conducted 6–17 october 2014,” 2014.

[6] E. Bonabeau, “Agent-based modeling: Methods and techniques for simulating human systems,” *Proceedings of the national academy of sciences*, vol. 99, no. suppl 3, pp. 7280–7287, 2002.

[7] T. E. C. Jones-Konneh, A. Murakami, H. Sasaki, and S. Egawa, “Intensive education of health care workers improves the outcome of ebola virus disease: Lessons learned from the 2014 outbreak in sierra leone,” *The Tohoku Journal of Experimental Medicine*, vol. 243, no. 2, pp. 101–105, 2017.

[8] International Organization for Migration, “Sierra leone summary sheet,” https://www.iom.int/countries/sierra-leone, 2015.

[9] J. Raven, H. Wurie, and S. Witter, “Health workers' experiences of coping with the ebola epidemic in sierra leone's health system: a qualitative study,” *Bmc Health Services Research*, vol. 18, no. 1, p. 251, 2018.

[10] A. A. Annan, D. D. Yar, M. Owusu, E. A. Biney, P. K. Forson, P. B. Okyere, A. A. Gyimah, and E. Owusu-Dabo, “Health care workers indicate ill preparedness for ebola virus disease outbreak in ashanti region of ghana,” *BMC Public Health*, vol. 17, no. 1, p. 546, 2017.

[11] The AnyLogic company, “Anylogic,” https://www.anylogic.cn/.

[12] R. Potluri, A. Kumar, V. Maheshwari, C. Smith, V. Oriol Mathieu, K. Luhn, B. Callendret, and H. Bhandari, “Impact of prophylactic vaccination strategies on ebola virus transmission: A modeling analysis,” *Plos one*, vol. 15, no. 4, p. e0230406, 2020.

[13] C. Siettos, C. Anastassopoulou, L. Russo, C. Grigoras, and E. Mylonakis, “Modeling the 2014 ebola virus epidemic–agent-based simulations, temporal analysis and future predictions for liberia and sierra leone,” *PLoS currents*, vol. 7, 2015.

[14] E. ça Suffit Ring Vaccination Trial Consortium, “The ring vaccination trial: a novel cluster randomised controlled trial design to evaluate vaccine efficacy and effectiveness during outbreaks, with special reference to ebola,” *BMJ: British Medical Journal*, vol. 351, p. h3740, 2015.

[15] A. M. Henao-Restrepo, I. M. Longini, M. Egger, N. E. Dean, W. J. Edmunds, A. Camacho, M. W. Carroll, M. Doumbia, B. Draguez, S. Duraffour *et al.*, “Efficacy and effectiveness of an rvsv-vectored vaccine expressing ebola surface glycoprotein: interim results from the guinea ring vaccination cluster-randomised trial,” *The Lancet*, vol. 386, no. 9996, pp. 857–866, 2015.

[16] W. H. Organization, “Weekly epidemiological record,” vol. 91, no. 14, pp. 181–192, 2016.
