# Supplementary material for: A hybrid simulation model to study the impact of combined interventions on Ebola epidemic
Source: PLoS One. 2021 Jul 6;16(7):e0254044. doi: 10.1371/journal.pone.0254044 (PMC8259970; doi:10.1371/journal.pone.0254044)
Supplement: Attachment — Submitted filename: Response to reviewers.pdf [file pone.0254044.s004.pdf]

Reply to the Editors Comments and Reviewers on Paper PONE-D-20-38313R1

Submission to PLOS ONE

June 7, 2021

1 Response to Editors

Thank you for your letter and for the reviewers' comments concerning our manuscript entitled "A hybrid simulation model to study the impact of combined interventions on Ebola epidemic" (PONE-D-20-38313R1). We appreciate the editors and reviewers for the time and effort that they have put into reviewing the previous version of the manuscript. Their suggestions have enabled us to improve our work. We have studied comments carefully and have made correction. The revised portion is marked in blue in the paper.

Appended to this letter is our point-by-point response to the comments raised by the reviewers. The comments are reproduced and our responses are given directly afterward. We would like to express our sincere thanks to you and reviewers for the constructive and positive comments and we hope that the revised manuscript is accepted for publication in PLOS ONE.

Yours Sincerely

Peiyu Chen

2 Response to Reviewer #1

Comment: work more on improving the quality of the figures (in the PDF version the quality of resolution is rather low) and importantly report and overlay the e.g. 95% confidence intervals in all relevant figures (figures for the cumulative ebola cases, deaths, etc, impact of pre-job training etc, impact of prophylactic vaccination.

Reply: Many thanks for this suggestion. We refreshed all the figures and checked with PACE which helps ensure that figures meet PLOS requirements. However, the figures shown in the preview pdf are still low in pixels. We ensure that the tiff files we uploaded meet the requirement and the figures can be seen in the tiff attachments. Beisides, we added the 95%CI for the modeling results in Fig 1 (fig 6 in the revised manuscript) in the part of model validation. However, we did not add 95%CI for figures about the impact of interventions, because the curves in the figures are fitted through some discrete points and we can only give 95%CI for those points. Those discrete points which have the same X-axis value are closed and their 95%CI have high overlap ratios which cannot be distinguished in the figures. Owing to that, we listed all the 95%CI for those points in the supplementary data(S1 File). And we also plot the statistical information for these points by boxplots in Fig 2 "The median, interquartile range and 1.5 times the interquartilerange of the simulation output under different combination of interventions" (fig 10 in the revised manuscript).

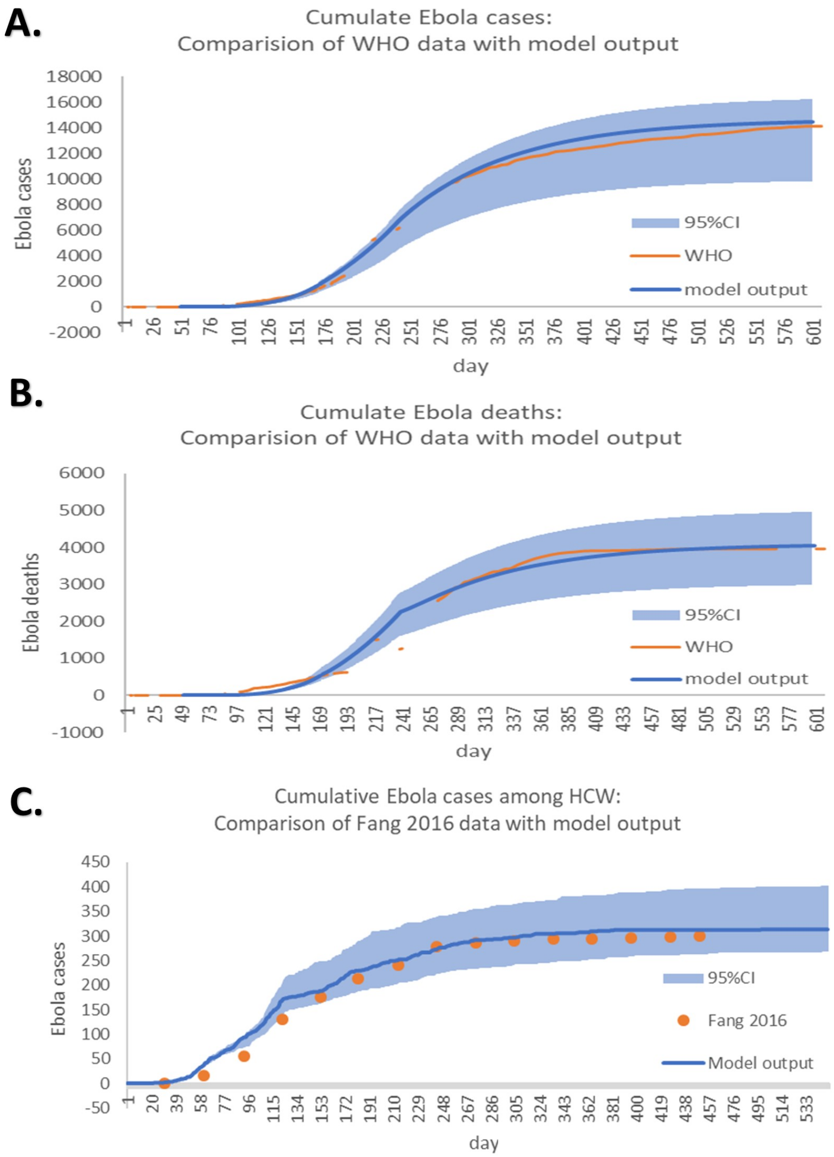

Fig. 1. Comparison of the output of the simulation result with published data.

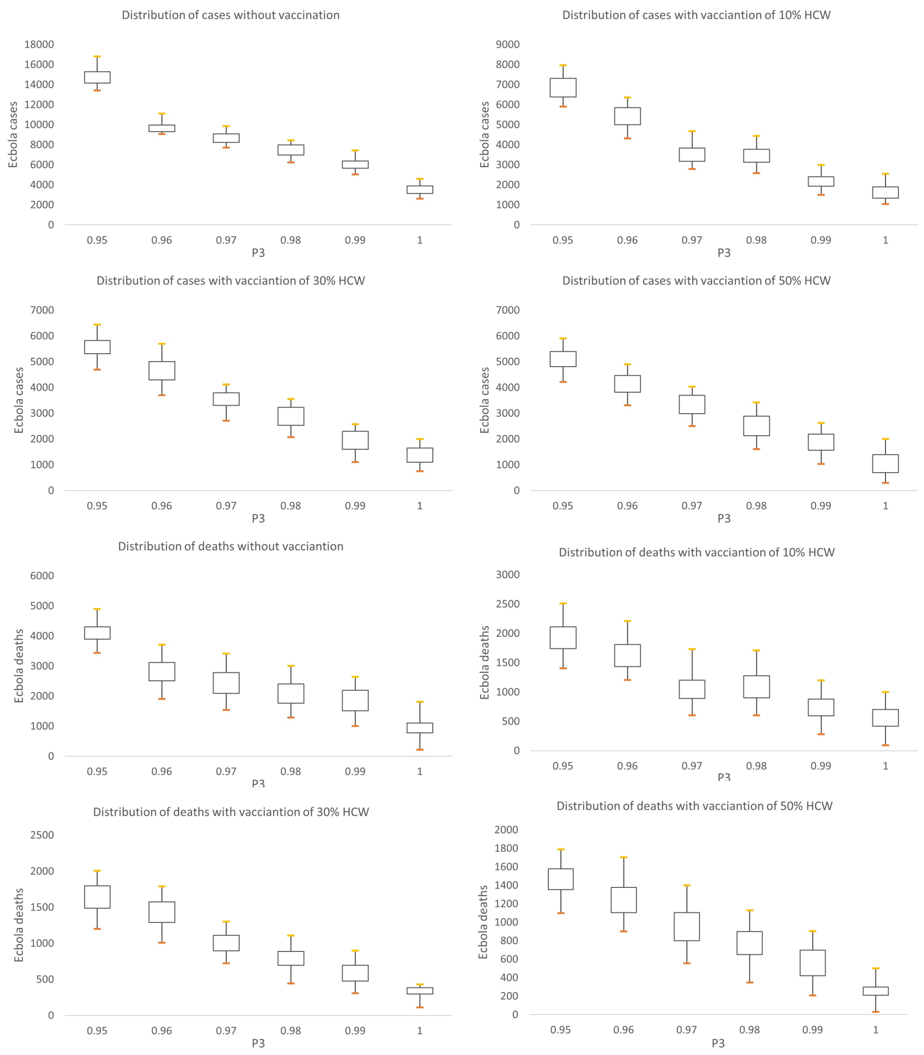

Fig. 2. The median, interquartile range and 1.5 times the interquartilerange of the simulation output under different combination of interventions

<u>*Comment:*</u> Please make clear, what data have been used for calibration: on the cumulative or the new cases (at least for the deaths and recovered)?. It is known that a calibration based on long series of cumulative data can bias the confidence intervals of estimations. So it is better that the calibration is made on the new cases (see also the discussion in King et al. Avoidable errors in the modelling of outbreaks of emerging pathogens, with special reference to ebola, Proceedings of the Royal Society B: Biological Sciences 282 (1806) (2015) 20150347.

<u>*Reply:*</u> Thanks for your insightful suggestion. As you stated, it is true that using the new cases is better than the cumulative cases. So we re-calibrated our model based on the daily new cases and deaths in the overall population. And we refreshed the model parameters (marked in blue in Table 1 in the revised manuscript), all the simulation results(Table 3-6 in the revised manuscript and the corresponding figures) and the modeling results in Table 1-2 in S1 file. The difference between the updated model and the previous model is slight. The following reasons will illustrate this result. Firstly, the use of agent-based modeling method increases the uncertainty of the result and the bias caused by cumulative data is relatively insignificant. Secondly, parameters in our model were referred to the parameters reported by Potluri R [1] which had been calibrated

already. Besides, we also described the data used for calibration in the revised manuscript(Line 261-262).

3 Response to Reviewer #2

Comment: The authors made sufficient efforts to address satisfactorily all my concerns. The paper is now very nice, well writing and suitable for publication.
Reply: Many thanks for your recognition of our work. And we really appreciate for your warm work.

References

[1] R. Potluri, A. Kumar, V. Maheshwari, C. Smith, V. Oriol Mathieu, K. Luhn, B. Callendret, and H. Bhandari, "Impact of prophylactic vaccination strategies on ebola virus transmission: A modeling analysis," *Plos one*, vol. 15, no. 4, p. e0230406, 2020.
